# Revealing the nature of optical activity in carbon dots produced from different chiral precursor molecules

**DOI:** 10.1038/s41377-022-00778-9

**Published:** 2022-04-11

**Authors:** Ananya Das, Evgeny V. Kundelev, Anna A. Vedernikova, Sergei A. Cherevkov, Denis V. Danilov, Aleksandra V. Koroleva, Evgeniy V. Zhizhin, Anton N. Tsypkin, Aleksandr P. Litvin, Alexander V. Baranov, Anatoly V. Fedorov, Elena V. Ushakova, Andrey L. Rogach

**Affiliations:** 1 Center of Information Optical Technologies, ITMO University, Saint Petersburg, 197101 Russia; 2 Research Park, Saint Petersburg State University, Saint Petersburg, 199034 Russia; 3 Laboratory of Femtosecond Optics and Femtotechnology, ITMO University, Saint Petersburg, 197101 Russia; 4 Laboratory of Quantum Processes and Measurements, ITMO University, Saint Petersburg, 197101 Russia; 5 Department of Materials Science and Engineering, and Centre for Functional Photonics (CFP), City University of Hong Kong, Kowloon, Hong Kong SAR, 999077 China; 6 Shenzhen Research Institute, City University of Hong Kong, Shenzhen, 518057 China

**Keywords:** Optical materials and structures, Circular dichroism

## Abstract

Carbon dots (CDs) are light-emitting nanoparticles that show great promise for applications in biology and medicine due to the ease of fabrication, biocompatibility, and attractive optical properties. Optical chirality, on the other hand, is an intrinsic feature inherent in many objects in nature, and it can play an important role in the formation of artificial complexes based on CDs that are implemented for enantiomer recognition, site-specific bonding, etc. We employed a one-step hydrothermal synthesis to produce chiral CDs from the commonly used precursors citric acid and ethylenediamine together with a set of different chiral precursors, namely, L-isomers of cysteine, glutathione, phenylglycine, and tryptophan. The resulting CDs consisted of O,N-doped (and also S-doped, in some cases) carbonized cores with surfaces rich in amide and hydroxyl groups; they exhibited high photoluminescence quantum yields reaching 57%, chiral optical signals in the UV and visible spectral regions, and two-photon absorption. Chiral signals of CDs were rather complex and originated from a combination of the chiral precursors attached to the CD surface, hybridization of lower-energy levels of chiral chromophores formed within CDs, and intrinsic chirality of the CD cores. Using DFT analysis, we showed how incorporation of the chiral precursors at the optical centers induced a strong response in their circular dichroism spectra. The optical characteristics of these CDs, which can easily be dispersed in solvents of different polarities, remained stable during pH changes in the environment and after UV exposure for more than 400 min, which opens a wide range of bio-applications.

## Abstract (web summary)

Using different chiral precursor molecules, we produced chiral CDs with superior optical properties, such as two-photon absorption, high photoluminescence quantum yield reaching 57%, and chiral signals in the UV-Vis spectral region.

## Graphical abstract

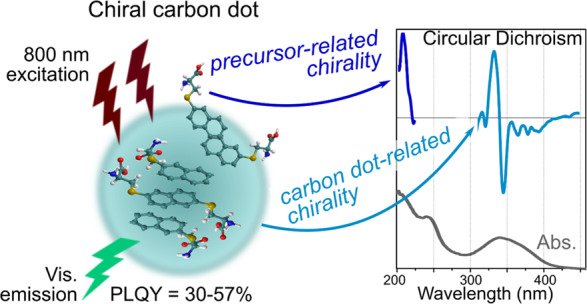

## Introduction

The development of nanomaterials for theranostics is an urgent task^1^. Ideal candidates should simultaneously serve as markers for early diagnosis of diseases and possess a therapeutic effect, and their structural parameters and physicochemical properties should be effectively tuned^2–4^. Luminescent carbon nanoparticles termed carbon dots (CDs) are promising in this respect, and this is due to their biocompatibility, bright emission, and ease of fabrication and further chemical functionalization^5–7^. Much work has been done on the development of synthetic protocols for highly emissive CDs with photoluminescence (PL) quantum yields (QYs) over 50%^8–12^, shifting their optical transitions to deep-red and near-infrared spectral regions by increasing size of *sp*^2^-domains^13^ and N-doping^14^. This is needed for improving the resolution and sensitivity of bioimaging^15–17^, as well as for widening the toolkit for functionalization of the CD surface, such as by attaching proteins and antibodies for targeted bonding to living tissues^18,19^, sensing ions^20^, and increasing photothermal conversion for cancer treatment^21^. Optical chirality, on the other hand, is an intrinsic property of many natural objects, which also attracts scientific attention for biomedical applications, such as enantioselective recognition and chiral sensing^22^. Chiral CDs can be formed on chiral substrates such as cellulose nanocrystals^23^ or produced by a one-pot synthesis from chiral precursors such as citric acid and d-proline^24^ or l/d-glutamine^25^. Another way to produce chiral CDs is postsynthetic treatment of achiral CDs with chiral molecules, including proline, phenylalanine, histidine, tryptophan, alanine, and proline methyl ester, as was shown by Ostadhossein et al.^26^, and tyrosine, phenylalanine, tryptophan, serine, and glutamic acid, as was shown by Zhang et al.^27^. In this case, the chirality of CDs is mainly inherited from chiral molecules. In our recent study^28^, we offered a comparison of these approaches and showed that surface functionalization and one-pot synthesis result in optical transitions in circular dichroism spectra of different natures, namely, inherited chirality from the precursor, hybridization of the energy levels of the precursor and CD, and formation of a chiral CD core. However, the origin of chirality in CDs produced by one-pot syntheses and the pathways for controlling the circular dichroism signal still require attention.

Another important feature that should be considered in bioimaging applications of CDs is improvement of the resolution and the signal-to-noise ratio by minimizing autofluorescence, which can be achieved by red-shifting the optical transitions into the deep-red and near-infrared spectral regions^13,20^, or multiphoton excitation of CD emission^29^. The latter approach offers reduced autofluorescence and a larger penetration depth, longer observation time, and less photodamage to biological tissues^30^. Thus, the development of synthetic methods for formation of chiral CDs with high multiphoton absorption cross-sections is of current interest, but it is challenging.

Herein, we developed synthetic routes toward chiral CDs from different chiral precursors. The resulting CDs possessed rather superior optical properties: their PLQYs reached 57%, they showed chiral signals in the UV and visible spectral regions, and the emission could be excited through two-photon absorption. Combined with the stable optical characteristics of these CDs, which did not change much upon variation of environmental pH and after UV exposure for more than 400 min, the chiral CDs produced in this work may find applications in bioimaging, sensing, drug delivery, and theranostics.

## Results

**Fig. 1:**
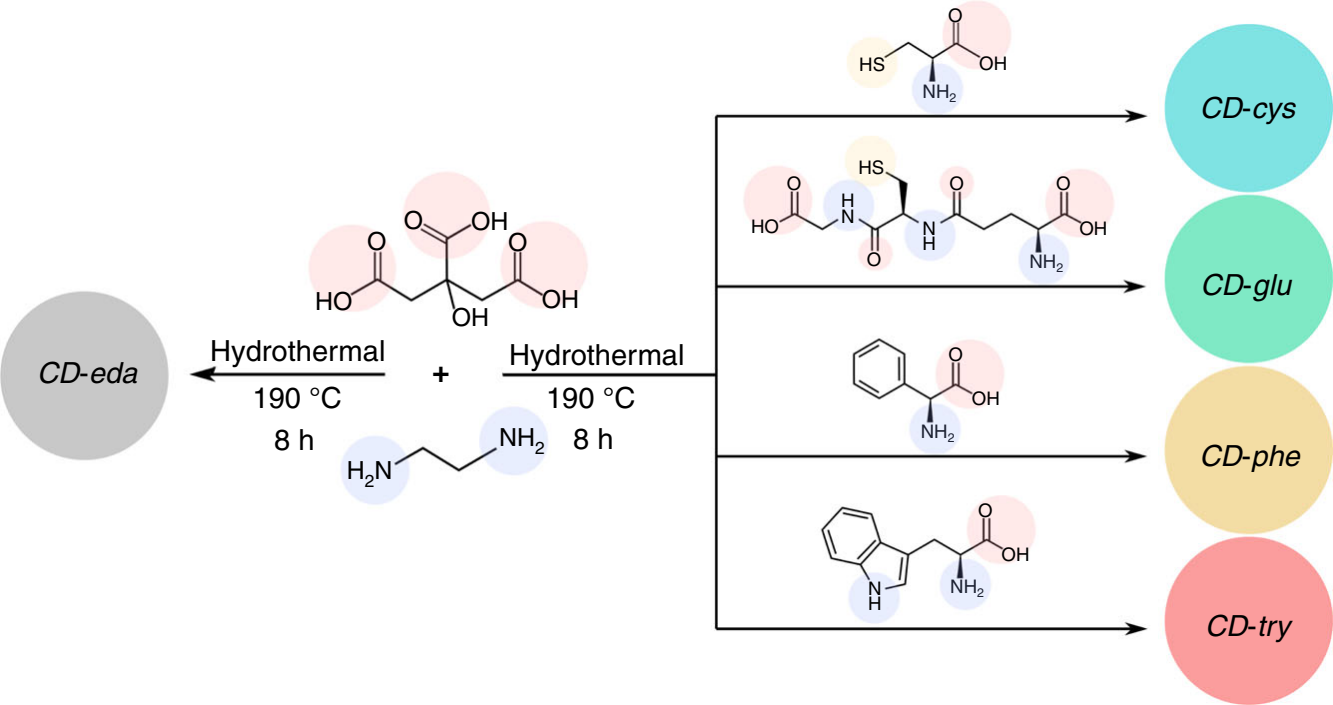
Syntheses of achiral CDs (*CD-eda*) and chiral CD samples (*CD-cys*, *CD-glu*, *CD-phe*, and *CD-try*). Common precursors citric acid and ethylenediamine were combined with a set of different chiral precursors, l-cysteine, l-glutathione, l-phenylglycine, and l-tryptophan. Chemical functional groups present in the precursor molecules, namely, carboxylic, amino, and thiol groups, are highlighted by blue, red, and yellow semitransparent circles, respectively

A set of four CDs was synthesized in water under hydrothermal conditions (190 °C, 8 h) from citric acid and ethylenediamine while varying the chiral precursor molecules l-cysteine, l-glutathione, l-phenylglycine, and l-tryptophan; they are designated hereafter as *CD-cys*, *CD-glu*, *CD-phe*, and *CD-try*, respectively (Fig. 1). Cysteine contains an α-amino group, an α-carboxylic acid group, and a methionine side chain. Glutathione is a well-known zwitterionic tripeptide consisting of glutamic acid attached via its side chain to the N-terminal cysteinyl glycine. Thus, both cysteine and glutathione contain sulfur as a heteroatom, in addition to nitrogen and oxygen. Phenylglycine contains a benzene ring bonded to the α-carbon as a side chain and tryptophan contains indole as a side chain attached to the β-carbon along with the α-amino group and α-carboxylic acid group. CDs hydrothermally synthesized under the same conditions from citric acid and ethylenediamine served as a reference sample and are designated *CD-eda* or “achiral CDs” in the forthcoming discussion. A detailed description of the synthetic procedures for all the samples is provided in the “Materials and methods” section.

### Size and morphology of CDs

**Fig. 2:**
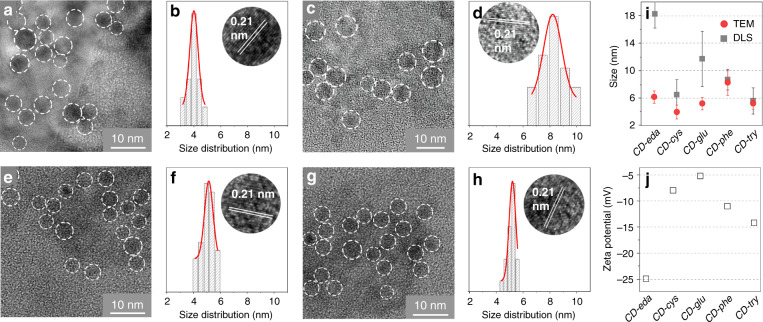
Morphologies of CD samples. *CD-cys* (**a**, **b**), *CD-glu* (**c**, **d**), *CD-phe* (**e**, **f**), *CD-try* (**g**, **h**). **a**, **c**, **e**, **g** TEM images; **b**, **d**, **f**, **h** size distribution histograms and HRTEM images (insets). **i** Comparison of CD sizes estimated from TEM images (red circles) and DLS measurements (gray squares). **j** Zeta potentials of CD samples

Transmission electron microscopy (TEM) images shown in Fig. 2 certify that all of the synthesized CDs comprise spherical nanoparticles. The size distribution was calculated from the TEM images by counting approximately 100 particles for every sample; the average sizes of CDs were found to be 4.0 ± 0.4, 5.2 ± 0.4, 8.2 ± 0.8, and 5.2 ± 0.3 nm for *CD-cys*, *CD-glu*, *CD-phe*, and *CD-try*_,_ respectively. The particle average size for the achiral *CD-eda* was determined to be 6.3 ± 0.4 nm (Fig. S1). Representative high-resolution TEM (HRTEM) images of single CDs shown in Fig. 2 revealed that all samples contained *sp*^2^-domains with an interplanar distance of 0.21 nm, which corresponds to the (100) crystal planes of graphitic carbon^31^. The hydrodynamic sizes of CDs have been estimated from dynamic light scattering (DLS), as shown in Fig. 2i. According to the DLS analysis, they were 18.2 ± 2.5, 6.5 ± 2.2, 11.7 ± 4.0, 8.7 ± 1.5, and 5.6 ± 1.9 nm for *CD-eda*, *CD-cys*, *CD-glu*, *CD-phe*, and *CD-try*_,_ respectively. From the comparison of CD sizes estimated from TEM and DLS measurements (Fig. 2i), it can be inferred that the increased DLS diameter for *CD-cys* and *CD-glu* may be related to different extension of the hydrated CD surface. Figure 2j shows the zeta potentials of the CDs, which certify that all of them possess a negatively charged surface (−8.0, −5.2, −11.0, and −14.2 mV for *CD-cys*, *CD-glu*, *CD-phe*, and *CD-try*, respectively). The zeta potential of achiral CD (*CD-eda*) showed the most negative charge among the samples (−25.0 mV), which may be related to its largest hydrodynamic size estimated from DLS measurements (Fig. 2i).

A comparison of TEM and DLS data suggests that the use of precursors containing benzene rings (*sp*^2^-hybridized domains), such as l-phenylglycine and l-tryptophan, results in the formation of CDs with smaller hydrodynamic sizes and surface groups with larger negative charges. The use of precursors with aliphatic chains, such as l-cysteine and l-glutathione, results in the formation of smaller particles, and the surface groups provide smaller negative charges.

### Chemical composition of CDs

**Fig. 3:**
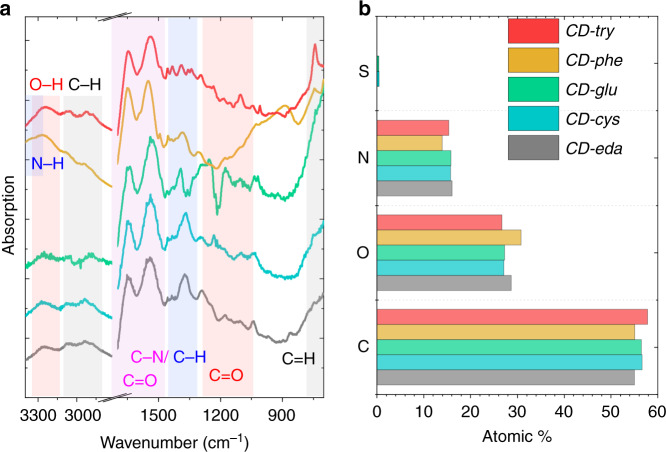
Chemical compositions of five CD samples. *CD-eda* (gray), *CD-cys* (cyan), *CD-glu* (green), *CD-phe* (orange), and *CD-try* (red). **a** FTIR spectra with characteristic peaks corresponding to different chemical bonds are highlighted in different colors; (**b**) atomic percentage of different chemical elements (C, O, N, S) constituting CDs, as derived from XPS measurements

Fourier transform infrared (FTIR) spectra of five CD samples are shown in Fig. 3a. All CDs had a broad and intense band at 3100–3500 cm^−1^, which was attributed to N–H groups and H-bonding of the –OH groups. Two distinct bands at ~3060 and 2880–2940 cm^−1^ were also observed for all CDs, and these were due to the presence of C–H stretching modes of aromatic and aliphatic carbons, respectively. These bands were more intense for *CD-phe* and *CD-try*. The most intense peaks for all CDs were observed at 1645 and 1535 cm^−1^ and were ascribed to –C=O stretching and N–H bending trans to the carbonyl oxygen of the amide group, respectively. In the spectral region 1500–1600 cm^−1^, bands corresponding to C=C stretching in benzene rings were observed for all samples. In addition to those peaks, *CD-phe* and *CD-try* exhibited a strong and narrow peak at 740 cm^−1^, which was attributed to a = C–H bending vibration in a benzene ring. For *CD-phe* and *CD-try*, a set of peaks in the range 1400–1450 cm^−1^ was observed and attributed to the C–N stretching mode of the amide group. Another intense peak associated with C–N stretching was observed at 1370 cm^−1^ for *CD-eda* and *CD-cys*, which was found at 1380 cm^−1^ for *CD-phe* and to 1390 cm^−1^ for *CD-glu* and *CD-try*. For all samples, the set of peaks in the region 1210–1300 cm^−1^ and peaks at 1180 and 1050 cm^−1^ were attributed to C–N and C–O stretching modes. Thus, we can conclude that all CD samples consisted of O, N-doped carbon networks rich in hydroxy and amide groups.

The chemical compositions of the five CD samples were further investigated by X-ray photoelectron spectroscopy (XPS). Full survey XPS spectra (Fig. S2) confirmed that all CDs were composed of C, O, and N atoms, whose relative atomic percentages are shown in Fig. 3b and in Table S1. Along with O and N atoms, *CD-cys* and *CD-glu* also contained 0.5 and 0.4% S atoms, respectively (Table S1). From the high-resolution XPS spectra provided in Fig. S3, the C 1*s* peak consisted of three peaks attributed to C–C/C=C (284.4 eV), C–O/C–N (286.2 eV), and C=O (288.4 eV) bonds. The integrated intensities of the C–C/C=C peak for *CD-cys*, *CD-glu*, and *CD-phe* were larger than those for *CD-eda* and *CD-try*. The N 1*s* peak consisted of two bands at 399.6 and 401.5 eV, which were attributed to pyrrolic N and amino groups, respectively. The O 1*s* peak consisted of two bands at ~531.2 and ~532.6 eV, which were attributed to C=O and C–O/O–H, respectively. For *CD-phe*, a peak at 532.8 eV, which corresponded to C–O/O–H bonds, dominated the O 1*s* spectrum. For the S 2*p* spectra of CD-cys and CD-glu, two peaks at approximately 164.2 and 168.4 eV were attributed to –SH and –SO_4_, respectively (Fig. S4).

The Raman spectra of the five CD samples contained four bands—D* or TPA, D, A, and G (Fig. S5), which could be attributed to a disordered graphitic lattice or *trans*-polyacetylene chains, breathing modes of *sp*^2^-domains, amorphous carbon with *sp*^3^-hybridization, and a stretching mode for *sp*^2^-domains, respectively^32^. Thus, all of these CD samples could be viewed as amorphous carbon matrices that included domains of *sp*^2^-hybridized carbon^33^. We can conclude that the synthesized CDs have O, N-doped (and for *CD-cys*, *CD-glu* also S-doped) carbon networks in their cores, amide and hydroxy groups at their surfaces, and rather similar chemical compositions.

### Optical properties of CDs

**Fig. 4:**
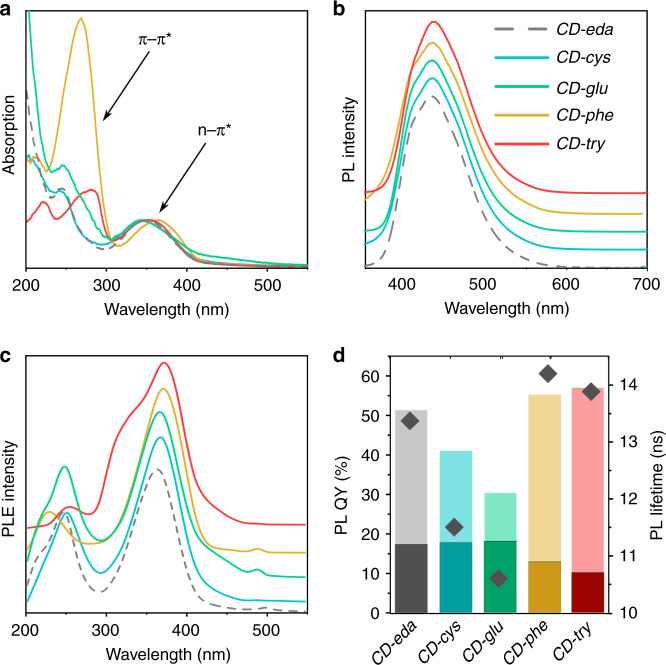
Optical properties of five CD samples. *CD-eda* (gray), *CD-cys* (cyan), *CD-glu* (green), *CD-phe* (orange), and *CD-try* (red). **a** Absorption spectra; **b** normalized PL spectra arbitrarily offset for clarity of presentation; the excitation wavelength was 350 nm; **c** normalized PLE spectra arbitrarily offset for clarity of presentation and monitored at 490 nm; **d** PLQYs (bars) of as-prepared (light color) and UV-exposed (dark color; see text) CDs, together with their average PL lifetimes (diamond symbols) in water. CDs were exposed to UV (*λ* = 366 nm) irradiation for 420 min

The optical characteristics of all CD samples synthesized in this work are summarized in Table S2. In the absorption spectra of the five CD samples shown in Fig. 4a, two distinct regions can be highlighted: one is between 200 and 300 nm and another is between 300 and 400 nm. Optical transitions in the 200–300 nm spectral region can be ascribed to π–π* transitions in *sp*^2^-hybridized carbon domains^29^. Peaks located at 210 and 245 nm in the absorption spectra were almost identical for *CD-eda*, *CD-cys*, and *CD-glu*. *CD-phe* also had a peak at 210 nm, but another peak was more intense and shifted to 270 nm. In the absorption spectrum of *CD-try*, these two peaks were further shifted toward the longer-wavelength region and were observed at 220 and 280 nm. The absorption band at 240–280 nm corresponding to amino acids may coincide with the π–π* transition of CDs^34^. The second region (300–400 nm) was commonly attributed in the literature to n–π* transitions^29^. In this spectral range, one broad absorption peak at 340 nm was observed for *CD-eda*, *CD-cys*, and *CD-glu*, and it was found at 350 and 365 nm for *CD-phe* and *CD-try*, respectively. These trends can be related to the combined effects of increased O-doping, carbonization, and size, especially for the *CD-phe* sample. Moreover, *CD-glu* showed increased absorption in the spectral region >450 nm, which was attributed to the presence of molecular groups on the surface.

The PL spectra of the five CD samples were almost the same, with a PL peak position at 450 nm independent of an excitation wavelength shorter than 400 nm (Fig. 4b and Fig. S6). When excited with wavelengths longer than 400 nm, the PL peak positions for all samples red-shifted as the excitation wavelength was increased (Fig. S6), which indicated the presence of lower energy states. PL excitation (PLE) spectra of the five CD samples monitored at the PL maximum (490 nm) are shown in Fig. 4c; they generally corresponded to the absorption spectrum for each respective sample. For *CD-eda*, *CD-cys*, and *CD-glu*, PLE peaks were observed at 245 nm with a shoulder at 215 and at 365 nm. For the other two chiral CDs, these peaks were red-shifted and observed at 230 and 370 nm for *CD-phe*, and at 250 and 370 nm (with a shoulder at 350 nm) for *CD-try*. Thus, the tendencies observed for the PL and PLE spectra suggested that emission originated primarily from n–π* transitions of similar molecular groups formed during CD syntheses and was independent of the type of chiral molecules used.

At the same time, the chemical compositions of the chiral precursors affected the PLQY and PL decay times, as shown in Fig. 4d. The PLQY of achiral *CD-eda* was 51%, while those for *CD-cys* and *CD-glu* decreased to 41% and 30%, respectively. For the *CD-phe* and *CD-try* samples synthesized in the presence of phenylglycine and tryptophan—two molecules containing benzene rings—the PLQYs reached 55% and 57%, respectively. The same trend was observed for the average PL lifetimes, which are shown with diamonds in Fig. 4d; these were derived from the PL decays shown in Fig. S7. The average PL lifetimes were 13.3, 11.5, 10.9, 14.2, and 13.9 ns for *CD-eda*, *CD-cys*, *CD-glu*, *CD-phe*, and *CD-try*, respectively. From the equation ${\mathrm {PLQY}} = \frac{\tau }{{\tau _{\mathrm r}}} = \frac{{k_{\mathrm r}}}{{k_r + k_{{\mathrm {nr}}}}}$, where *τ* is the measured PL lifetime, *τ*_r_ is the natural or intrinsic lifetime, and *k*_r_ and *k*_nr_ are the emissive and nonradiative decay rates of the fluorophore, respectively, the latter parameters were estimated and are listed in Table S2. These parameters were compared with those of fluorophores often formed during CD syntheses and exhibiting similar optical properties^35^, namely, blue-emissive citrazinic acid and 1,2,3,5-tetrahydro-5-oxo-imidazo[1,2-a]pyridine-7-carboxylic acid (IPCA). It is worth mentioning that citrazinic acid and IPCA exhibit spectral parameters similar to those of the synthesized CDs, as shown in Table S2. The CDs synthesized in this work demonstrated slightly increased intrinsic PL lifetimes within the range of 24–36 ns, compared to 20 and 17 ns for citrazinic acid^36^ and IPCA^37^, respectively (Table S2). This can be explained by the interactions of luminescence centers within CDs that resulted in increases in PL lifetimes together with decreases in PLQYs, as was recently shown for acridone derivatives^38^.

### Effects of UV exposure, pH, and solvent polarity on the optical properties of CDs

For potential applications of CDs, it is important to determine the stability of their emission under continuous UV excitation; details of the experiments are provided in the Supporting Information. The changes in PL intensities of the samples shown in Fig. S8 together with the changes in PLQYs (shown in Fig. 4d) after 420 min of UV exposure suggest that the photostabilities of CDs were inversely proportional to the initial PLQYs. This can be related to the photobleaching rates of emissive states within CDs: molecular states with higher PLQYs have lower stabilities and are more prone to damage by photobleaching, as shown in several studies^39^. Figure S8a shows that stronger declines in PL intensities, especially for *CD-glu* and *CD-try*, were observed during the first 50 min of UV irradiation, after which they remained rather stable. After 420 min under UV exposure, the PL intensities decreased to 60%, 43%, 33%, 23%, and 18% of the initial PL intensities for *CD-glu*, *CD-cys*, *CD-eda*, *CD-phe*, and *CD-try*, respectively (Fig. S8a). The difference in photostabilities of chiral CDs compared to achiral CDs is also reflected by changes in the shapes of the PL bands, as shown in Fig. S8b. This was attributed to the different photostabilities of luminescence centers within CDs, in particular a possible difference between core-related and surface-related emissions^40^, which was also reflected by the differences in the absorption and PLE spectra of *CD-cys* and *CD-glu* compared to *CD-phe* and *CD-try* (Fig. 4a, c). The photobleaching process was irreversible for all studied samples. After UV irradiation for 420 min, the PLQYs of *CD-eda*, *CD-cys*, *CD-glu*, *CD-phe*, and *CD-try* were still 17%, 18%, 13%, 10%, and 18%, respectively (Fig. 4d), which makes the implementation of such CDs as luminescent agents in bioimaging rather feasible^41^.

The effect of pH on the optical properties of the chiral CDs produced here was studied for the CDs with the highest PLQY, namely, *CD-phe*. The pH of the initial aqueous solution of these CDs was approximately 6, which was changed to become more acidic (down to 0) or more basic (up to 12) by addition of concentrated HCl or KOH, respectively (see details in Supporting Information). Within this rather broad range of pH values, the PL intensity of the CDs was only reduced by approximately 15–25%, and even for a strongly acidic pH of 0, the reduction was rather moderate at approximately 40% (Fig. S9a). The PL spectral profile also remained largely unaltered over the whole pH range from 0 to 12 (Fig. S9a). PLQYs remained in the range 48% (pH 0) to 55% (pH 6) to 52% (pH 12) (Fig. S9b). The same trend was observed for the average PL lifetimes (Fig. S9b), which varied within the range 8.6–14.2 ns, indicating that the ratios of *k*_r_ to *k*_nr_ remained almost unchanged and pH changes did not affect the relaxation process of the *CD-phe* emissive state (Table S3).

Investigations of the optical characteristics of CDs dispersed in solvents with different polarities were conducted for *CD-phe* and *CD-try* (see details in Supporting Information). For both *CD-phe* and *CD-try*, the absorption peaks for the bands attributed to n–π* transitions were blue-shifted when they were dispersed in less polar solvents (from water to toluene), as shown in Fig. S10a, d. This suggests that the solvent polarity had little effect on the ground states of CDs. More pronounced changes were observed for their excited states: the solvent dielectric constant affected both the PL peak position and the overall shape of the PL band, as shown in Fig. S10b, e for *CD-phe* and *CD-try*, respectively. The PL band excited at 350 nm blue-shifted from 450 to 417 nm and from 447 to 403 nm as the solvent dielectric constant decreased from that of water to that of toluene for *CD-phe* (Fig. S10a) and *CD-try* (Fig. S10d), respectively. We noticed that the observed blue-shift of the PL peak with decreasing solvent polarity has been reported previously for CDs^33^ and ascribed to general solvent effects, including solvent relaxation depending on the refractive index and dielectric constant^42^. At the same time, for both *CD-phe* and *CD-try* in protic polar solvents (alcohols and water), the PL bands were red-shifted by approximately 10–20 nm (Fig. S10a, d) compared to those in aprotic solvents (acetone, acetonitrile). Moreover, the overall shapes of the PL bands of CDs reflect contributions from multiple emissive centers with different natures existing simultaneously in CDs^29,33^, and upon decreasing the solvent polarity (going from water to toluene), the blue component became more intense compared to the longer-wavelength emission (Fig. S10a, d). This observation suggests that along with solvent relaxation, some specific solvent effects that are determined by the chemical properties of the emissive centers and solvent, such as protonation/deprotonation and hydrogen bonding, affect the excited state of CDs.

The average PL lifetimes of CDs also varied significantly in solvents of different polarities, as shown in Fig. S10c, f for *CD-phe* and *CD-try*, respectively. The maximal PL lifetimes for both samples were observed in water, which is the most polar solvent; they decreased from 14.2 to 5.5 ns for *CD-phe* and from 13.9 to 4.4 ns for *CD-try* upon changing the dielectric constant from 80.4 (water) to 2.3 (toluene). The PL lifetime increased linearly with increasing dielectric constant of the solvent, indicating an increase in the ratio of *k*_r_ to *k*_nr_ with increasing solvent polarity, as was shown for amino groups containing coumarin-151 (ref. ^43^). It should be noted that, following the differences in PL band positions (Fig. S10a, d) for both *CD-phe* and *CD-try*, the average PL lifetimes for CDs dispersed in protic polar solvents were 1–2 ns longer compared to those in aprotic solvents (Fig. S10c, f), suggesting that protonation of the excited state not only affected energy levels but also relaxation rates. This dependence is potentially useful for fluorescence lifetime imaging during changes in the environment in living tissues, both in terms of pH^44^ and solvent polarity values^45^.

### Demonstration of CD chirality

**Fig. 5:**
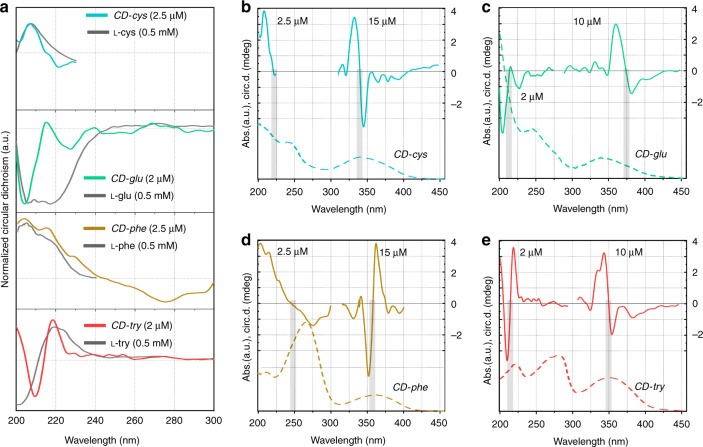
Chiroptical properties of CDs. **a** Comparison of normalized circular dichroism spectra of the four precursor molecules (gray lines) with those of the chiral CDs in the UV spectral region; concentrations of CDs and their precursors are given in brackets. The dashed line shows the zero level. **b**–**e** Absorption spectra (dashed lines) and circular dichroism (solid lines) spectra of four chiral CD samples: **b** *CD-cys*, **c** *CD-glu*, **d** *CD-phe*, and **e** *CD-try*. The values of the circular dichroism signals are given in *mdeg* on the right side of each graph. Gray rectangles emphasize the sign change in the circular dichroism spectra. Concentrations of CDs for particular regions of the circular dichroism spectra are given in **b–e** for clarity (see text for details)

Circular dichroism spectroscopy is a form of light absorption spectroscopy that measures the difference in absorbance of right- and left-circularly polarized light for a studied material. This method is useful for analyzing the absolute configurations and conformations of chiral compounds. All four CDs synthesized from the chiral precursors showed nonzero signals in their circular dichroism spectra (Fig. 5); this was different for achiral *CD-eda*, for which this signal was absent (Fig. S11). It is worth mentioning that the chiral CDs samples were thoroughly purified to avoid any influence of residual nonreacted chiral precursors left in solution. To obtain a stronger signal, different concentrations of CDs and their precursors were used for each particular case, as specified hereafter. In Fig. 5a, we compare the circular dichroism spectra of chiral CDs with those of their chiral precursors in the spectral range 200–300 nm. Their signals appeared to be rather similar in the 200–240 nm spectral region, with some alterations depending on the types of chiral CDs. For *CD-cys* (concentration 2.5 μM), the peak at 210 nm almost coincided with that of the l-cysteine precursor (concentration 0.5 mM). For *CD-glu* (2 μM), the circular dichroism spectrum showed two minima at 205 and 225 nm, which were situated within the location of the broad and strong peak of their precursor l-glutathione (0.5 mM). For the *CD-phe* (2.5 μM) and l-phenylglycine (0.25 mM) solutions, the circular dichroism spectra were similar in the 200–240 nm region and showed several peaks. To check whether the negative broad peak at 275 nm originated from aggregates of precursor molecules, a 5 mM l-phenylglycine solution was also measured and showed a peak at 260 nm (Fig. S12). Such a red-shift from 260 to 275 nm could be due to absorption flattening and circular differential scattering, which are mainly observed for aggregates^46–48^. This observation suggests that the circular dichroism signal in the 240–300 nm spectral region may have originated from hybridization of lower-energy levels of chiral chromophores in aggregates attached to the CD surfaces or formed within the CDs. For *CD-try* (2 μM), the circular dichroism spectrum was also quite similar to that of the precursor l-tryptophan, but the peaks observed at 210 and 218 nm were slightly red-shifted. From these observations, we can conclude that the chiral signals observed in the 200–300 nm region for the CDs originated from the intrinsic chirality of their precursors, which sometimes coincided with (or became dominated by) signals from aggregates that formed both at the surface or within the respective CDs. To further confirm this assumption, an effect of pH on circular dichroism signals was studied for *CD-phe* and its precursor, l-phenylglycine (Fig. S13). For a decrease in pH down to pH = 1, an increase in the amplitude of the circular dichroism signal at 260 nm was observed and attributed to aggregation of l-phenylglycine. Hence, in *CD-phe* circular dichroism spectrum, the appearance of a maximum at 260–270 nm with decreasing pH can be attributed to increased interactions of chiral chromophores aggregated at the CD surface. In addition, the change in the sign of the signal indicated a change in the mutual arrangement of chiral group axes in aggregates^49^.

To collect circular dichroism spectra in the 300–450 nm spectral region, the concentrations of CDs were increased to 15 μM for *CD-cys* and *CD-phe* and to 10 μM for *CD-glu* and *CD-try*. A comparison of the absorption and circular dichroism spectra of chiral CDs is provided in Fig. 5b–e. For *CD-cys*, two intense extrema at 332 and 345 nm were observed, with circular dichroism signals of 3.32 and −3.6 mdeg, respectively (Fig. 5b). Along with these intense peaks, weaker peaks appeared at 315, 365, and 380 nm with circular dichroism signals of 0.36, −0.98, and −0.94, respectively. All these peaks were observed within the *CD-cys* n–π* absorption band, with a sign switch between the most intense peaks corresponding to the absorption maximum at 340 nm. Such circular dichroism signals (bisignate intense peaks) were also observed for nanoparticles^50^ and organic molecules with two chiral chromophore groups^51^ and are referred to as exciton coupling^52^. For a pair of chiral chromophores, their interaction results in a split of an excited level, whereas the ground state remains unchanged, as shown in Fig. S14a. This exciton interaction or exciton coupling generates two electronic transitions: from the ground state to α- and β-polarized excited states with opposite rotational strengths. In circular dichroism spectra, the appearance of intense extrema/peaks with opposite signs, which are referred to as bisignate Cotton effect (CE)^53^, is shown schematically in Fig. S14b. We will use the term CE for designation of two intense peaks with a sign change in the chiral signal at the absorption maximum. Thus, since CE correspond to n–π* optical transitions of the CDs, we assumed that the chirality originated from interactions of chiral chromophores existing within CDs.

**Fig. 6:**
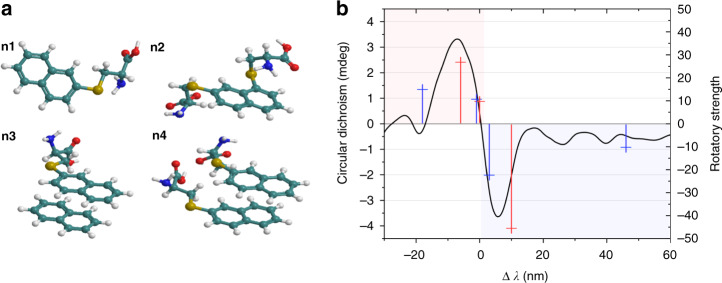
Theoretical modeling of the optical response for *CD-cys*. **a** Illustration of surface-located chiral optical centers of CDs based on a naphthol molecule with one (n1) or two (n2) l-cysteine molecules and a naphthol dimer with one (n3) or two (n4) l-cysteine molecules. These four models have been used to demonstrate the possible chiral features of *CD-cys* in the longer-wavelength region 300–400 nm. **b** Comparison of the calculated rotary strength for n3 (red crosses) and n4 (blue crosses) and the measured circular dichroism spectrum for *CD-cys* at the wavelength scale relative to the maximum absorption bands at 309, 318, and 340 nm for (n3), (n4), and *CD-cys*. In (**b**), positive second Cotton and negative first Cotton effects are highlighted by red and blue rectangles, respectively

To further understand the nature of the circular dichroism signal in the longer-wavelength spectral region, in particular, the existence of several extrema within the CD absorption band and the reason for the changes in their signs, we carried out theoretical modeling of the optical response for *CD-cys*. We analyzed the impacts of interactions among optical centers (chromophores) typical for CDs and chiral precursors on the circular dichroism spectra of CDs by using a quantum–chemical approach (see “Materials and methods” section for details). As illustrated in Fig. 6a, we considered four possible configurations of the surface optical centers and the chiral precursors: a center representing a polycyclic aromatic hydrocarbon (PAH) covalently linked to one (n1) or two (n2) l-cysteines and a center representing a noncovalent PAH dimer with one (n3) or two (n4) l-cysteines. In the calculations performed, we assumed the PAH optical centers to be composed of naphthol molecules. Table S4 summarizes the wavelengths, oscillator strengths, and rotatory strengths of the lowest-energy absorption bands of the naphthol-based surface optical centers of *CD-cys* obtained from DFT calculations. The surface optical center with a chiral l-cysteine molecule (n1) showed one absorption band at 313 nm in the longer-wavelength region with a negative sign for rotatory strength. Two l-cysteine molecules attached to the naphthol-based surface optical center (n2) induced negative and positive CEs for the absorption bands at 318 and 344 nm, respectively. The surface center in the form of a noncovalent naphthol dimer with one l-cysteine molecule had three CEs at 303, 309, and 319 nm with the sign pattern (+), (+), and (−) for the rotatory strength. Two l-cysteine molecules attached to the dimer of the naphthol-based surface optical center (n4) gave rise to four CEs at 300, 317, 321, and 364 nm and a sign pattern for the rotatory strength of (+), (+), (−), and (−). Thus, our simulations demonstrated the possibility of various sign patterns for the rotatory strength in the longer-wavelength region of the circular dichroism spectra. To qualitatively compare the model with our experimental data, we plotted the rotatory strengths of the calculated structures (n3 and n4) together with the *CD-cys* circular dichroism spectrum using the wavelength scale relative to the position of the absorption maximum, Δ*λ* (Fig. 6b). The sign patterns for the (n3) and (n4) surface configurations are in reasonable qualitative agreement with the experimental data, as shown in Fig. 6b. Moreover, the calculated parameters for circular polarization of the structures consisting of only one naphthol molecule—(n1) and (n2)—matched the *CD-cys* circular dichroism spectrum poorly (Fig. S15). Thus, longer-wavelength chiral signals (300–400 nm) were attributed to interactions of chiral chromophores with PAH aggregates (dimers) of different configurations formed within the CD structure, including the chiral cores of CDs.

Similar chiral signals were observed for all other chiral samples; the positions of their extrema and amplitudes of their circular dichroism signals are summarized in Table S5. Compared to the other chiral CDs described in this work, the CEs for *CD-glu* were red-shifted to 362 and 383 nm compared to the absorption band maximum at 340 nm, which can be attributed to the difference between absorptions of chiral chromophores and nonchiral compounds existing in the CD structure. Another interesting feature was observed for *CD-phe*, for which CEs exhibited the opposite sign pattern and the circular dichroism values were −4.6 mdeg at 352 nm and 3.8 mdeg at 362 nm. It is worth noting that the sign pattern of the CEs (Fig. S14b) depends on the mutual arrangement of two long axes of chiral chromophores^51^. A comparison of circular dichroism spectra obtained under different pH conditions for *CD-phe* in the 300–400 nm spectral region, which were attributed to n–π* optical transitions of the CDs, revealed that the red-shifts of CEs accompanied an absorption peak red-shift (Fig. S16). This observation further confirmed that the circular dichroism signals in the longer-wavelength region originated from the interactions of chiral chromophores with PAH molecules/aggregates formed within the CD structure.

A quantitative analysis of the chiroptical properties of CDs was conducted by calculating the dissymmetry factor (*g*-factor) as $(A_{\mathrm L} - A_{\mathrm R})/A$, where *A*_L_ and *A*_R_ are the absorbance of left- and right-circularly polarized light, respectively, and *A* is the absorbance of unpolarized light. The *g*-factors corresponding to absorption at different wavelengths are summarized in Table S5. The *g*-factor varied from 10^–5^ to 10^–3^ depending on the sample and the spectral region, i.e., whether it was attributed to π–π* or n–π* optical transitions. Compared to those in our previous work, the *g*-factor was improved by one order of magnitude, and for the best sample *CD-phe*, it reached 1.6 × 10^–3^ and −4.1 × 10^–5^ for peaks at 225 and 352 nm, respectively. These values are comparable with those for allenoacetylenic dimers (approx. −2.5 × 10^–3^ at 210 nm)^54^, organic emissive molecules (1.5 × 10^−4^ at 390 nm)^55^, and even CdSe quantum rods (4.5 × 10^−4^ at 580 nm)^56^ and CdSe nanoplatelets (−6.7 × 10^−4^ at 500 nm)^57^.

It is worth mentioning that the optical activities of all chiral CDs in the 200–300 nm spectral region remained stable during storage under ambient conditions for up to 6 months, while the peaks observed at 300–400 nm showed a change in sign. A similar change in the sign of the circular dichroism signal was observed for chiral CDs synthesized from l- or d-glutathione in aqueous alkali with increasing electrolysis time^58^. Since circular dichroism can provide evidence of conformational changes in chiral chromophores, including self-assembly of superstructures^51,59^, we speculate that the sign change observed here may have originated from displacement of chromophoric groups related to n–π* optical transitions. A detailed study of this effect will be a subject of forthcoming work.

### Two-photon absorption and emission of CDs

Emission excited through multiphoton absorption is an attractive feature of several kinds of fluorophores and light-emitting nanoparticles and can be implemented in bioimaging and sensing to avoid the autofluorescence of tissues^17,41^. To date, several groups have reported CDs with PL occurring through two-photon absorption^30,60,61^ or even three-photon absorption^62^, which were used for two-photon cellular imaging under near-infrared laser excitation. In our previous related work, we showed that the two-photon absorption cross-sections of chiral CDs can reach 13 GM, which is comparable to those of organic dyes^28^.

**Fig. 7:**
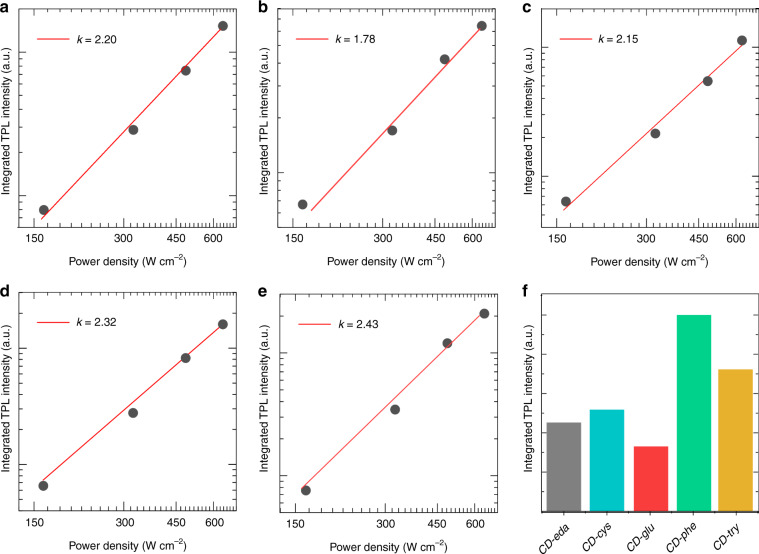
Two-photon excited luminescence of CDs. **a–e** Dependence of the integrated intensities of TPL on the power densities for excitation of the five CD samples: **a** *CD-eda*, **b** *CD-cys*, **c** *CD-glu*, **d** *CD-phe*, and **e** *CD-try*. **f** Comparison of integrated TPL intensities for five CD samples

All five CDs synthesized in this work demonstrated two-photon excited luminescence (TPL) signals when excited by an 800 nm femtosecond laser (see “Materials and methods” section for details). Their integrated TPL intensities were linearly dependent on the excitation power density on a log-log scale, with slope values varying from 1.8 to 2.4 (Fig. 7a–e). This suggested that the emission was caused by a two-photon absorption process rather than one-photon absorption. A comparison of integrated TPL intensities (Fig. 7f) indicated that *CD-phe* showed the brightest TPL. We estimated two-photon absorption cross-sections (*σ*) for the five CD samples and summarized them in Table S6. The *σ* values varied from 39 to 141 GM and were larger than that for the molecular dye rhodamine 6G (33 GM), which was measured under the same excitation conditions. The largest *σ* value of 141 GM was observed for *CD-glu*, although this sample had the lowest PLQY (30%). As the multiphoton absorption cross-section depends on a variety of factors, such as the excitation source (wavelength, pulse width)^63^, chemical environment^64,65^, and types of luminescent centers^66^, further studies are needed to better understand the nature of the two-photon absorption/emission processes in CDs.

## Discussion

To conclude, we synthesized a set of chiral CDs by using common precursors citric acid and ethylenediamine with addition of L-isomers of four different molecules—cysteine, glutathione, phenylglycine, and tryptophan. Hydrothermally produced CDs were spherical nanoparticles with sizes up to 8.2 nm, which consisted of O,N-doped (and sometimes included S) carbonized cores and surfaces rich in amide and hydroxy groups. The absorption and PL spectra of these CDs were rather similar to those of achiral CDs and were governed by optical centers formed by dehydration of the carboxylic and amine groups of the precursors; the PL band was centered at 450–470 nm, and the PLQY reached 57%. CDs synthesized by using chiral molecules with *sp*^2^-conjugated systems, such as phenylglycine and tryptophan, exhibited PLQY values higher than 55%. Chiral signals for CDs were rather complex and originated from (i) the presence of chiral precursors attached to the CD surface, which were observed in the 200–250 nm spectral region; (ii) hybridization of lower-energy levels of chiral chromophores in aggregates formed at the surfaces of or within CDs, with chiral signals observed in the 250–300 nm spectral region; and (iii) interactions of chiral chromophores with PAHs and aggregates with different configurations formed within CDs, which can be attributed to formation of the CDs’ chiral core. Based on a DFT analysis, we showed how incorporation of the chiral precursors at the optical centers induced strong responses in their circular dichroism spectra. Moreover, emission excited by two-photon absorption was observed for all chiral CDs, and the absorption cross-sections of this emission were comparable to or larger than those of organic dyes. The optical responses of these CDs, which were easily dispersed in solvents of different polarities, remained stable during pH changes in the environment and after UV exposure for more than 400 min, which opens up a wide range of applications in bioimaging, sensing, and theranostics.

## Materials and methods

### Synthesis of carbon dots

All samples were synthesized by a hydrothermal method using citric acid and ethylenediamine and the addition of chiral molecules. To avoid contributions from unreacted chiral precursors, CDs were carefully purified by dialysis over a membrane with a pore size of 12 kDa for 2 days against deionized water and subsequent centrifugation. Details of the syntheses and chemicals used are provided in the Supporting Information.

### Methods

XPS measurements were performed on an Escalab 250Xi photoelectron spectrometer (Thermo Fisher Scientific) with Al Kα radiation (photon energy 1486.6 eV). Measurements were performed in the constant pass energy mode at 100 eV for the survey XPS spectra and at 50 eV for the core level spectra of single elements using an XPS spot size of 650 μm. Absorption and PL spectra were collected on a UV-3600 spectrophotometer (Shimadzu) and a spectrofluorimeter FP-8200 (Jasco), respectively. FTIR spectra were collected on a Tenzor II infrared spectrophotometer (Bruker). Time-resolved PL measurements were performed using a MicroTime 100 confocal microscope (PicoQuant) equipped with a ×3 objective (NA = 0.1) and a 405 nm pulsed diode laser. Circular dichroism absorption spectra were collected on a J-1500 (Jasco) spectrophotometer. Two-photon absorption measurements were performed with a 35 fs Ti:sapphire laser system (Avesta) with *λ* = 800 nm operated at 1 kHz, and the power densities varied from 150 to 650 W cm^−^^2^. The excitation light was focused onto a quartz cuvette using a 20 cm lens positioned 17 cm from the focus. The emission was focused by a lens with a focal length of 3 cm on a USB4000-UV-VIS-ES spectrophotometer (Ocean Optics).

In theoretical modeling, the optical centers of CDs were subjected to ground-state geometry optimizations within the DFT framework with the B3LYP functional^67^, empirical dispersion correction (DFT-D4) by Grimme and co-workers^68^, and the standard DZP Slater-type basis set^69^. Energies, oscillator strengths, and rotatory strengths were calculated using the TD-DFT approach with the B3LYP functional, dispersion correction (DFT-D4), and the DZP basis set. All calculations were performed using the Amsterdam density functional (ADF) package^70^.

## Supplementary information

Supporting Information

## References

[1] Lim EK (2015). Nanomaterials for theranostics: recent advances and future challenges. Chem. Rev..

[2] Jiang YY (2019). A generic approach towards afterglow luminescent nanoparticles for ultrasensitive in vivo imaging. Nat. Commun..

[3] Gauger AJ, Hershberger KK, Bronstein LM (2020). Theranostics based on magnetic nanoparticles and polymers: intelligent design for efficient diagnostics and therapy. Front. Chem..

[4] De M, Ghosh PS, Rotello VM (2008). Applications of nanoparticles in biology. Adv. Mater..

[5] Du JJ (2019). Carbon dots for in vivo bioimaging and theranostics. Small.

[6] Ross S (2020). The analytical and biomedical applications of carbon dots and their future theranostic potential: a review. J. Food Drug Anal..

[7] Li WD (2019). Kilogram-scale synthesis of carbon quantum dots for hydrogen evolution, sensing and bioimaging. Chin. Chem. Lett..

[8] Tao SY (2020). Crosslink-enhanced emission effect on luminescence in polymers: advances and perspectives. Angew. Chem..

[9] Wang SC (2020). Enhanced-fluorescent imaging and targeted therapy of liver cancer using highly luminescent carbon dots-conjugated foliate. Mater. Sci. Eng. C.

[10] Wang JJ (2020). Highly luminescent copper iodide cluster based inks with photoluminescence quantum efficiency exceeding 98%. J. Am. Chem. Soc..

[11] Das A (2018). Carbon dot with pH independent near-unity photoluminescence quantum yield in an aqueous medium: electrostatics-induced Forster resonance energy transfer at submicromolar concentration. J. Phys. Chem. Lett..

[12] Das A (2017). On the molecular origin of photoluminescence of nonblinking carbon dot. J. Phys. Chem. C..

[13] Wang BY (2020). Rational design of multi-color-emissive carbon dots in a single reaction system by hydrothermal. Adv. Sci..

[14] Yang X (2021). Red-emitting, self-oxidizing carbon dots for the preparation of white LEDs with super-high color rendering index. Sci. China Chem..

[15] Shi XX (2019). Far-red to near-infrared carbon dots: preparation and applications in biotechnology. Small.

[16] Zhou B (2019). Recent insights into near-infrared light-responsive carbon dots for bioimaging and cancer phototherapy. Inorg. Chem. Front..

[17] Li D (2021). Optical properties of carbon dots in the deep-red to near-infrared region are attractive for biomedical applications. Small.

[18] Dhenadhayalan N, Lin KC, Saleh TA (2020). Recent advances in functionalized carbon dots toward the design of efficient materials for sensing and catalysis applications. Small.

[19] Ali H, Ghosh S, Jana NR (2020). Fluorescent carbon dots as intracellular imaging probes. Wiley Interdiscip. Rev. Nanomed. Nanobiotechnol..

[20] Wang BY (2019). Near-infrared emissive carbon dots with 33.96% emission in aqueous solution for cellular sensing and light-emitting diodes. Sci. Bull..

[21] Li BL (2021). Recent advances and prospects of carbon dots in phototherapy. Chem. Eng. J..

[22] Kuznetsova V (2021). Ligand-induced chirality and optical activity in semiconductor nanocrystals: theory and applications. Nanophotonics.

[23] Chekini M (2020). Chiral carbon dots synthesized on cellulose nanocrystals. Adv. Opt. Mater..

[24] Liu S (2021). One-step hydrothermal synthesis of chiral carbon dots with high asymmetric catalytic activity for an enantioselective direct aldol reaction. Chem. Commun..

[25] Ma WY (2021). Photoluminescent chiral carbon dots derived from glutamine. Chin. Chem. Lett..

[26] Ostadhossein F (2018). Chirality inversion on the carbon dot surface via covalent surface conjugation of cyclic α-amino acid capping agents. Bioconjugate Chem..

[27] Zhang ML (2021). Chiral control of carbon dots via surface modification for tuning the enzymatic activity of glucose oxidase. ACS Appl. Mater. Interfaces.

[28] Das A (2021). Chiral carbon dots based on L/D-cysteine produced via room temperature surface modification and one-pot carbonization. Nanoscale.

[29] Ragazzon G (2021). Optical processes in carbon nanocolloids. Chem.

[30] Liu YF (2020). Rational synthesis of highly efficient ultra-narrow red-emitting carbon quantum dots for NIR-II two-photon bioimaging. Nanoscale.

[31] Wang ZW (2004). A quenchable superhard carbon phase synthesized by cold compression of carbon nanotubes. Proc. Natl Acad. Sci. USA.

[32] Ferrari AC, Robertson J (2000). Interpretation of Raman spectra of disordered and amorphous carbon. Phys. Rev. B.

[33] Stepanidenko EA (2020). Influence of the solvent environment on luminescent centers within carbon dots. Nanoscale.

[34] Pandit S (2019). In situ synthesis of amino acid functionalized carbon dots with tunable properties and their biological applications. ACS Appl. Bio Mater..

[35] Xiong Y (2018). Influence of molecular fluorophores on the research field of chemically synthesized carbon dots. Nano Today.

[36] Schneider J (2017). Molecular fluorescence in citric acid-based carbon dots. J. Phys. Chem. C.

[37] Song YB (2015). Investigation from chemical structure to photoluminescent mechanism: a type of carbon dots from the pyrolysis of citric acid and an amine. J. Mater. Chem. C.

[38] Liu RF, Zhu GX, Zhang G (2020). N-substitution of acridone with electron-donating groups: crystal packing, intramolecular charge transfer and tuneable aggregation induced emission. RSC Adv..

[39] Xiong Y (2017). Carbonization conditions influence the emission characteristics and the stability against photobleaching of nitrogen doped carbon dots. Nanoscale.

[40] Sun MH (2019). Realization of the photostable intrinsic core emission from carbon dots through surface deoxidation by ultraviolet irradiation. J. Phys. Chem. Lett..

[41] Su W (2020). Carbon dots: a booming material for biomedical applications. Mater. Chem. Front..

[42] Lakowicz, J. R. *Principles of Fluorescence Spectroscopy*. 3rd edn (Springer, 2006).

[43] Nad S, Pal H (2001). Unusual photophysical properties of coumarin-151. J. Phys. Chem. A.

[44] Yi ZH (2021). High quantum yield photoluminescent N-doped carbon dots for switch sensing and imaging. Talanta.

[45] Wang H (2020). Wide emission shifts and high quantum yields of solvatochromic carbon dots with rich pyrrolic nitrogen. Nano Res..

[46] Phillips CL (1986). Circular differential scattering and circular differential absorption of DNA-protein condensates and of dyes bound to DNA-protein condensates. Biochemistry.

[47] Castiglioni E (2007). Wavelength shifts in solid-state circular dichroism spectra: a possible explanation. Chirality.

[48] Deka MJ, Chowdhury D (2017). Chiral carbon dots and their effect on the optical properties of photosensitizers. RSC Adv..

[49] Harada N, Berova N (2012). 8.24 spectroscopic analysis: exciton circular dichroism for chiral analysis. Compr. Chirality.

[50] Ma W (2017). Chiral inorganic nanostructures. Chem. Rev..

[51] Berova, N., Ellestad, G. A., & Harada, N. In *Comprehensive Chiroptical Spectroscopy, Volume 1: Instrumentation, Methodologies, and Theoretical Simulations (eds Berova N., Polavarapu**P. L.**, Nakanishi**K.**, Woody**R. W.**)* Ch. 8.24 (Elsevier, 2012).

[52] Superchi S, Giorgio E, Rosini C (2004). Structural determinations by circular dichroism spectra analysis using coupled oscillator methods: an update of the applications of the DeVoe polarizability model. Chirality.

[53] Ben-Moshe A (2016). Probing the interaction of quantum dots with chiral capping molecules using circular dichroism spectroscopy. Nano Lett..

[54] Rivera-Fuentes P (2010). Amplification of chirality in monodisperse, enantiopure alleno-acetylenic oligomers. Angew. Chem. Int. Ed..

[55] Ma K (2019). Boosting the circularly polarized luminescence of small organic molecules via multi-dimensional morphology control. Chem. Sci..

[56] Gao XQ (2017). Excitonic circular dichroism of chiral quantum rods. J. Am. Chem. Soc..

[57] Gao XQ (2018). Distinct excitonic circular dichroism between wurtzite and zincblende CdSe nanoplatelets. Nano Lett..

[58] Zhang ML (2019). Maltase decorated by chiral carbon dots with inhibited enzyme activity for glucose level control. Small.

[59] Rodger A, Marshall D (2021). Beginners guide to circular dichroism. Biochemist.

[60] Liu KK (2019). Efficient red/near-infrared-emissive carbon nanodots with multiphoton excited upconversion fluorescence. Adv. Sci..

[61] Lan MH (2017). Two-photon-excited near-infrared emissive carbon dots as multifunctional agents for fluorescence imaging and photothermal therapy. Nano Res..

[62] Li D (2018). Near-infrared excitation/emission and multiphoton-induced fluorescence of carbon dots. Adv. Mater..

[63] Xu C, Webb WW (1996). Measurement of two-photon excitation cross sections of molecular fluorophores with data from 690 to 1050 nm. J. Optical Soc. Am. B.

[64] Nag A, Goswami D (2009). Solvent effect on two-photon absorption and fluorescence of rhodamine dyes. J. Photochem. Photobiol. A Chem..

[65] Zhang YL (2015). Solvent effect and two-photon optical properties of triphenylamine-based donor–acceptor fluorophores. J. Phys. Chem. C.

[66] Zhang Q (2017). Lighting the way to see inside two-photon absorption materials: structure–property relationship and biological imaging. Materials.

[67] Becke AD (1993). Density-functional thermochemistry. III. The role of exact exchange. J. Chem. Phys..

[68] Caldeweyher E (2019). A generally applicable atomic-charge dependent London dispersion correction. J. Chem. Phys..

[69] Van Lenthe E, Baerends EJ (2003). Optimized Slater-type basis sets for the elements 1-118. J. Comput. Chem..

[70] Te Velde G (2001). Chemistry with ADF. J. Comput. Chem..

