# Supplementary material for: Revealing the nature of optical activity in carbon dots produced from different chiral precursor molecules
Source: Light Sci Appl. 2022 Apr 11;11:92. doi: 10.1038/s41377-022-00778-9 (PMC9001697; doi:10.1038/s41377-022-00778-9)
Supplement: Supplementary file 1 — Supporting Information [file 41377_2022_778_MOESM1_ESM.docx]

Supporting Information for

**Revealing the nature of optical activity in carbon dots produced from different chiral precursor molecules**

Ananya Das,^a*^ Evgeny V. Kundelev,^a^ Anna A. Vedernikova,^a^ Sergei A. Cherevkov,^a^ Denis V. Danilov,^b^ Aleksandra V. Koroleva,^b^ Evgeniy V. Zhizhin,^b^ Anton N. Tsypkin,^c^ Aleksandr P. Litvin,^a,d^ Alexander V. Baranov,^a^ Anatoly V. Fedorov,^a^ Elena V. Ushakova,^a*^ and Andrey L. Rogach^e,f^

*^a^ Center of Information Optical Technologies, ITMO University, Saint Petersburg, 197101 Russia. E-mail: dasananyadas@gmail.com,* [*elena.ushakova@itmo.ru*](mailto:elena.ushakova@itmo.ru)

*^b^ Research Park, Saint Petersburg State University, Saint Petersburg, 199034 Russia*

*^c^ Laboratory of Femtosecond optics and Femtotechnology, ITMO University, Saint Petersburg, 197101 Russia*

*^d^ Laboratory of Quantum Processes and Measurements, ITMO University, Saint Petersburg, 197101 Russia*

*^e^ Department of Materials Science and Engineering, and Centre for Functional Photonics (CFP), City University of Hong Kong, Kowloon, Hong Kong, 999077, China*

*^f^ Shenzhen Research Institute, City University of Hong Kong, Shenzhen, 518057, China*

*Materials.* Citric acid (≥99.5 %), ethylenediamine (≥99.5 %), L-Cysteine (≥98.5 %), L-glutathione (≥98.5 %), L-Phenylglycine (≥99%) and L-Tryptophan (≥98.5 %), toluene, ethyl acetate, acetonitrile, 1-butanol, methanol were purchased from Sigma-Aldrich. All chemical reagents were used as received. Ultrapure (Milli-Q) water was used throughout experiments.

*Synthesis of achiral carbon dots (CD-eda).* 1g of citric acid and 1.01 mL of ethylenediamine (molar ratio 1:0.33) were dissolved in 10 mL of water and transferred into a Teflon-lined autoclave. The mixture was heated at 190^o^C for 8 h followed by solution purification via dialysis (membrane pore size 12 kDa) for two days against water, and subsequent centrifugation.

*Synthesis of chiral carbon dots (CD-cys, CD-glu, CD-phe, CD-try).* 1g of citric acid and 1.01 mL of ethylenediamine, 0.605 g of L-cystein (molar ratio of L-cysteine 1:1) were dissolved in 10 mL of water and transferred into a Teflon-lined autoclave. The mixture was heated at 190^o^C for 8 h, followed by the same purification procedure as for *CD-eda*. *CD-glu*, *CD-phe*, and *CD-try* were synthesized similarly to *CD-cys* maintaining the same molar ratio of citric acid and chiral precursor equal to 1:1. Thus, 1.54 g of L-glutathione, 0.756 g of L-phenylglycine, or 1.02 g of L-tryptophan were used to produce *CD-glu*, *CD-phe*, and *CD-try*, respectively.

*UV exposure*. We exposed aqueous solutions of CDs to UV lamp irradiation (366 nm wavelength) with a power of 1.4 W/m^2^. To avoid any direct heating, the illuminated cuvettes were placed into a water bath with water changed every 30 min. After each exposure time period, the CDs sample was taken out from water bath for spectral measurements, then put back for the UV exposure.

*pH adjustment*. pH value of original *CD-phe* aqueous solution was close to neutral (pH 6). 40, 60, and 80 μL of 0.1M KOH was added to 3 mL of *CD-phe* aqueous solution with an optical density of 0.1 to adjust the pH value to 9, 11, and 12, respectively. 40, 70, and 150 μL of 0.5M HCl was added to 3 mL *CD-phe* aqueous solution with an optical density of 0.1 to adjust the pH value to 2, 1, and 0, respectively.

*Optical characterization of CDs in solvents with different polarity.* CDs were dispersed in the following solvents with dielectric constant given in brackets: nonpolar solvents as toluene (2.3) and chloroform (9.1), dipolar aprotic as ethyl acetate (6.0), acetone (20.7) and acetonitrile (37.5), polar protic solvents including alcohols – butanol (17.1), isopropanol (18.3), ethanol (24.3), methanol (32.6), and water (80.4). It should be noted that, *CD-cys* and *CD-glu* were almost insoluble in nonpolar solvents.

*Calculation methods.* PLQY was estimated by a relative method; both the sample in question and the reference sample (quinine sulfate) were excited at 350 nm. PLQY was calculated using the following equation: $QY={QY}_{R}\frac{{OD}_{R}}{OD}\cdot\frac{I}{I_{R}}\cdot\frac{n^{2}}{n_{R}^{2}}$, where *QY*, *OD*, *I*, and *n* stands for quantum yield, optical density, integrated PL intensity and refractive index of the solvent, respectively; subscript *R* designates the same parameters for the reference sample. PL decay curves were fitted by a biexponential function: $I\left( t \right)=I_{0}+A_{1}e^{-t/\tau_{1}}+A_{2}e^{-t/\tau_{2}}$. The average PL lifetime was calculated as $\left\langle\tau\right\rangle={\sum A_{i}{\tau_{i}}^{2}}/{\sum A_{i}\tau_{i}}$. Two-photon absorption cross-section was calculated using following equations:$\sigma_{s}=\sigma_{r}\frac{\varphi_{r}}{\varphi_{s}}\frac{I_{s}^{\text{2P}}}{I_{r}^{\text{2P}}}\frac{C_{r}}{C_{s}}\frac{n_{r}}{n_{s}}$and $\varphi_{s}=\varphi_{r}\frac{I_{s}^{\text{1P}}}{I_{r}^{\text{1P}}}\frac{D_{r}}{D_{s}}\left( \frac{n_{r}}{n_{s}} \right)^{2}$, with *σ* ‑ cross section area, *ϕ* ‑ quantum yield, *I* ‑ fluorescence intensity, *C* ‑ concentration, *n* ‑ refractive index, *D* ‑ optical density; indexes *r* and *s* are for reference and investigated samples, respectively. Concentration of CDs was estimated using fluorescence correlation spectroscopy (FCS) implemented in a MicroTime 100 (PicoQuant). The FCS data were fitted by pure diffusion model in IGOR software: $G\left( \right)= \frac{1}{N} \frac{1}{1+(\frac{}{{}_{D}})} \surd\frac{1}{1+(\frac{}{{}_{D}})(\frac{1}{k^{2}})}$, with *G(τ)* ‑ auto correlation function, *N* ‑ average number of particles, *τ_D_* ‑ time of diffusion, *k* – structure parameter. After fitting, the average number of particles has been determined and CD concentration was calculated.


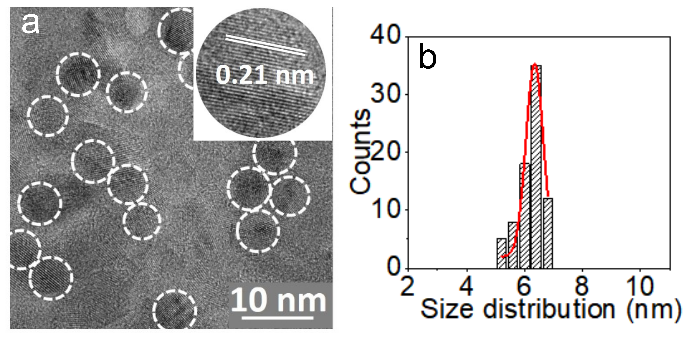


Figure S1. (a) TEM image of *CD-eda*; inset shows a HRTEM image of a single CD. (b) Size histogram of *CD-eda* determined from TEM images.


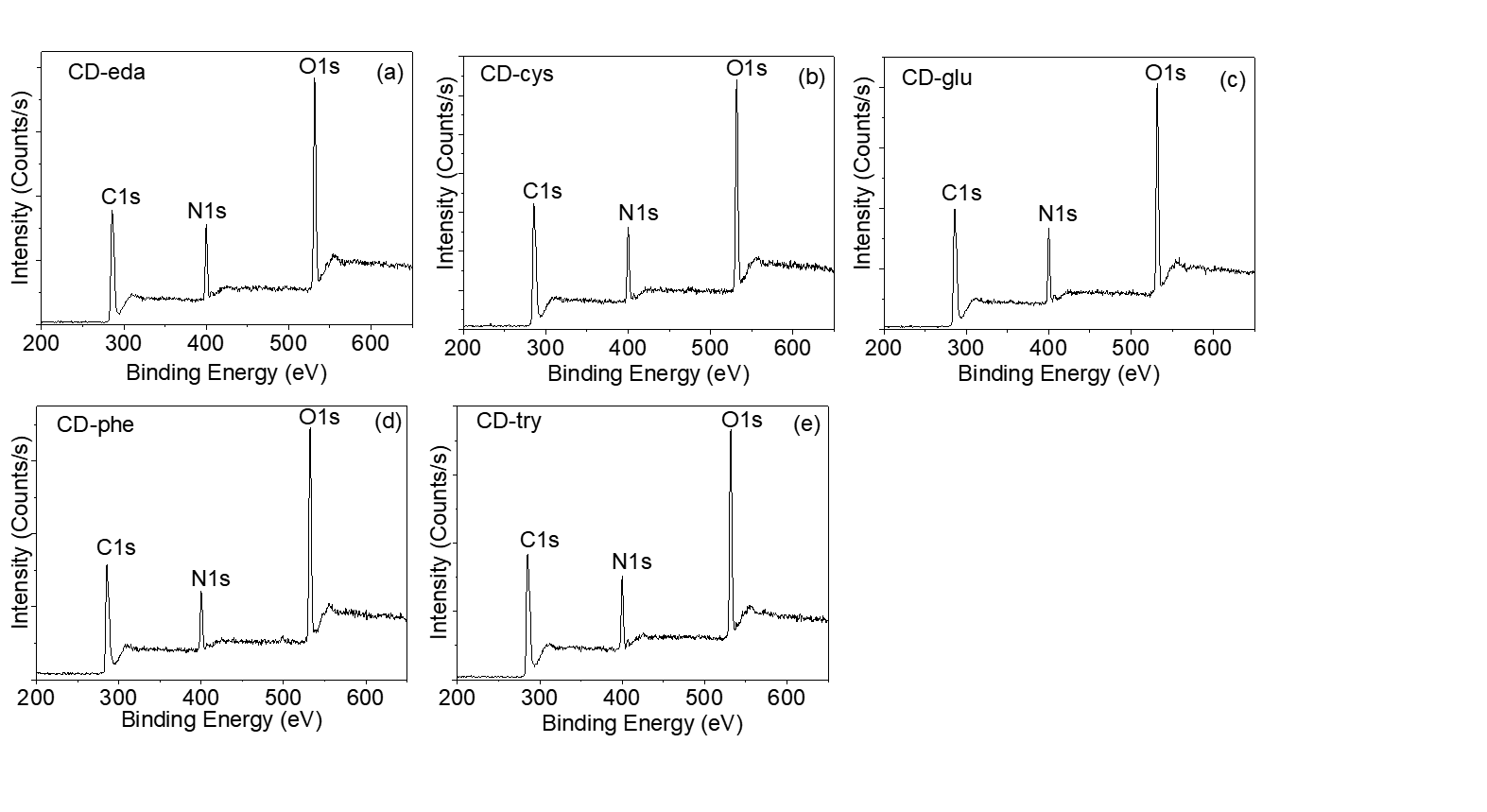


Figure S2. Full survey XPS spectra of five CD samples: (a) *CD-eda*, (b) *CD-cys*, (c) *CD-glu*, (d) *CD-phe*, and (e) *CD-try*.


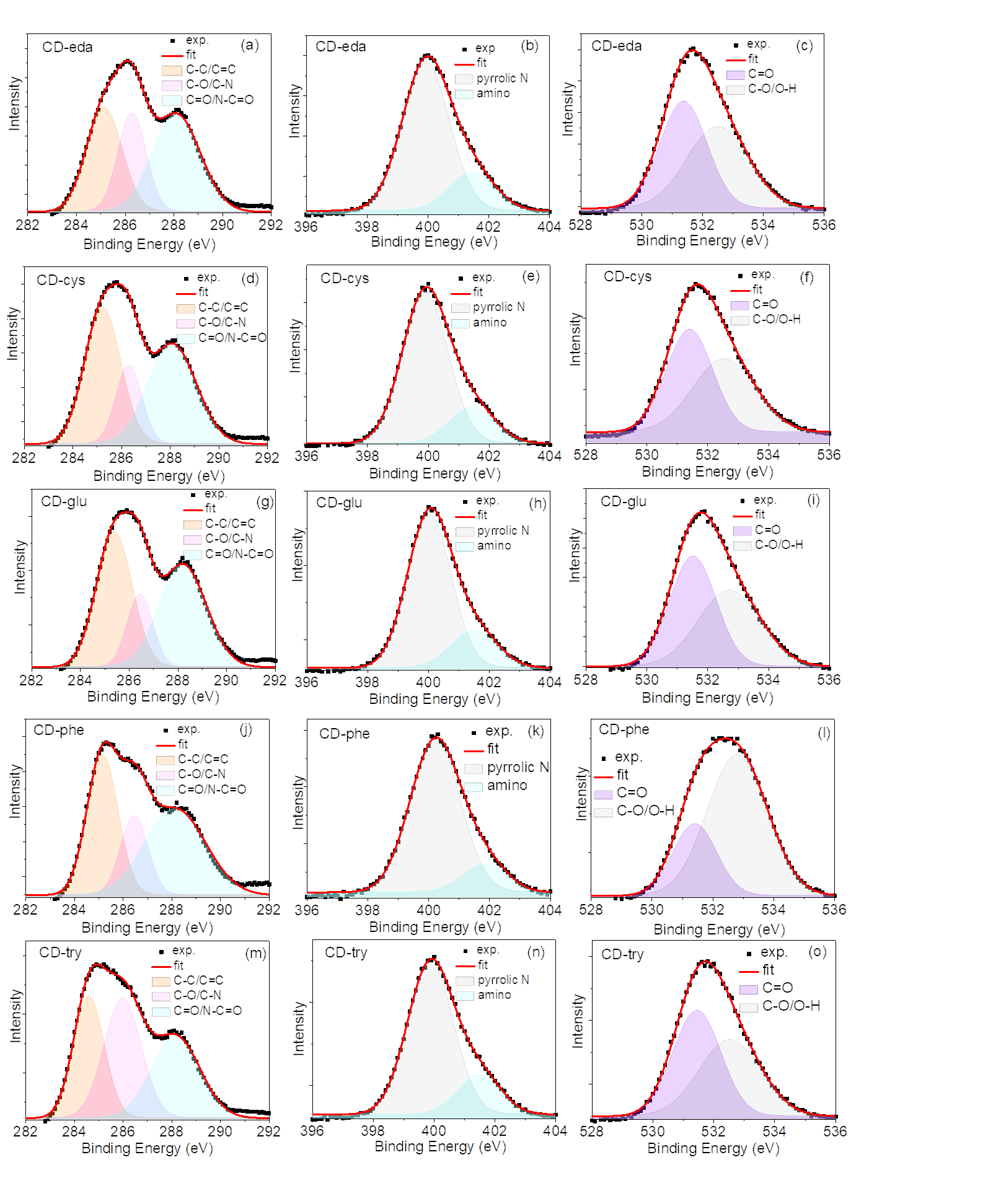


Figure S3. High-resolution XPS spectra of C 1S (first column: frames a, d, g, j, m), N 1S (second column: frames b, e, h, k, n), O 1S (third column: frames c, f, i, l, o) of five CD samples: *CD-eda* (a-c), *CD-cys* (d-f); *CD-glu* (g-i), *CD-phe* (j-l); *CD-try* (m-o). Experimental data (exp.) are shown by black dots, and the overall fitting curves (fit) – by red lines. Differently coloured individual deconvoluted peaks are assigned to different bonds as explained in the respective legends.


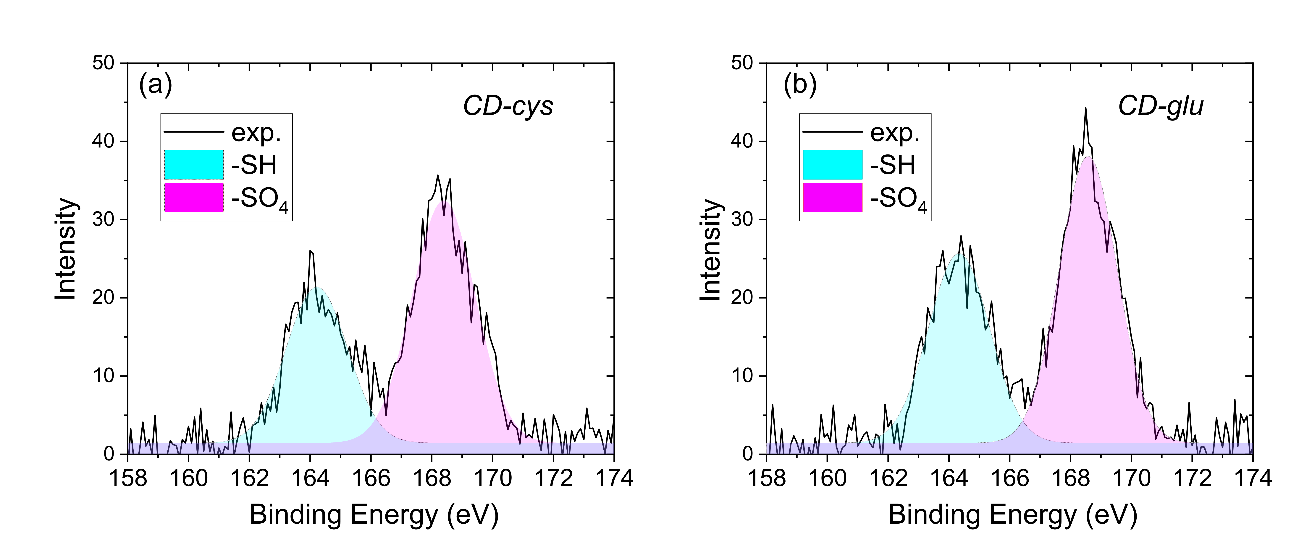


Figure S4. High-resolution XPS spectra of S 2p of CD-cys (a) and CD-glu (b). Experimental data (exp.) are shown by black lines, differently coloured individual deconvoluted peaks are assigned to different bonds as explained in the respective legends.


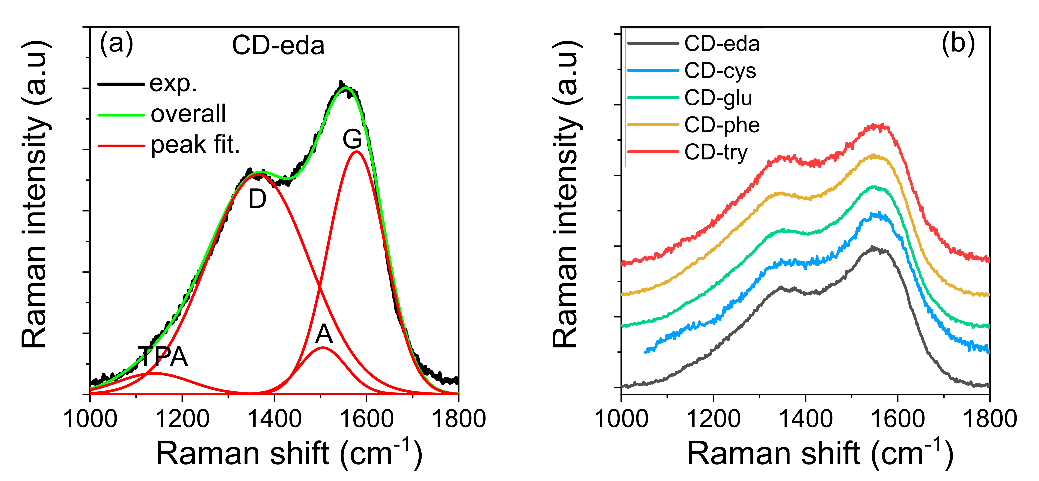


Figure S5. (a) Raman spectrum of *CD-eda*: experimental data are shown in black, deconvoluted peaks corresponding to TPA (D*), D, A, and G bands – in red, and the overall fitting curve – in green. (b) Normalized Raman spectra (arbitrary off-set for the clarity of presentation) of five CD samples listed in the legend.


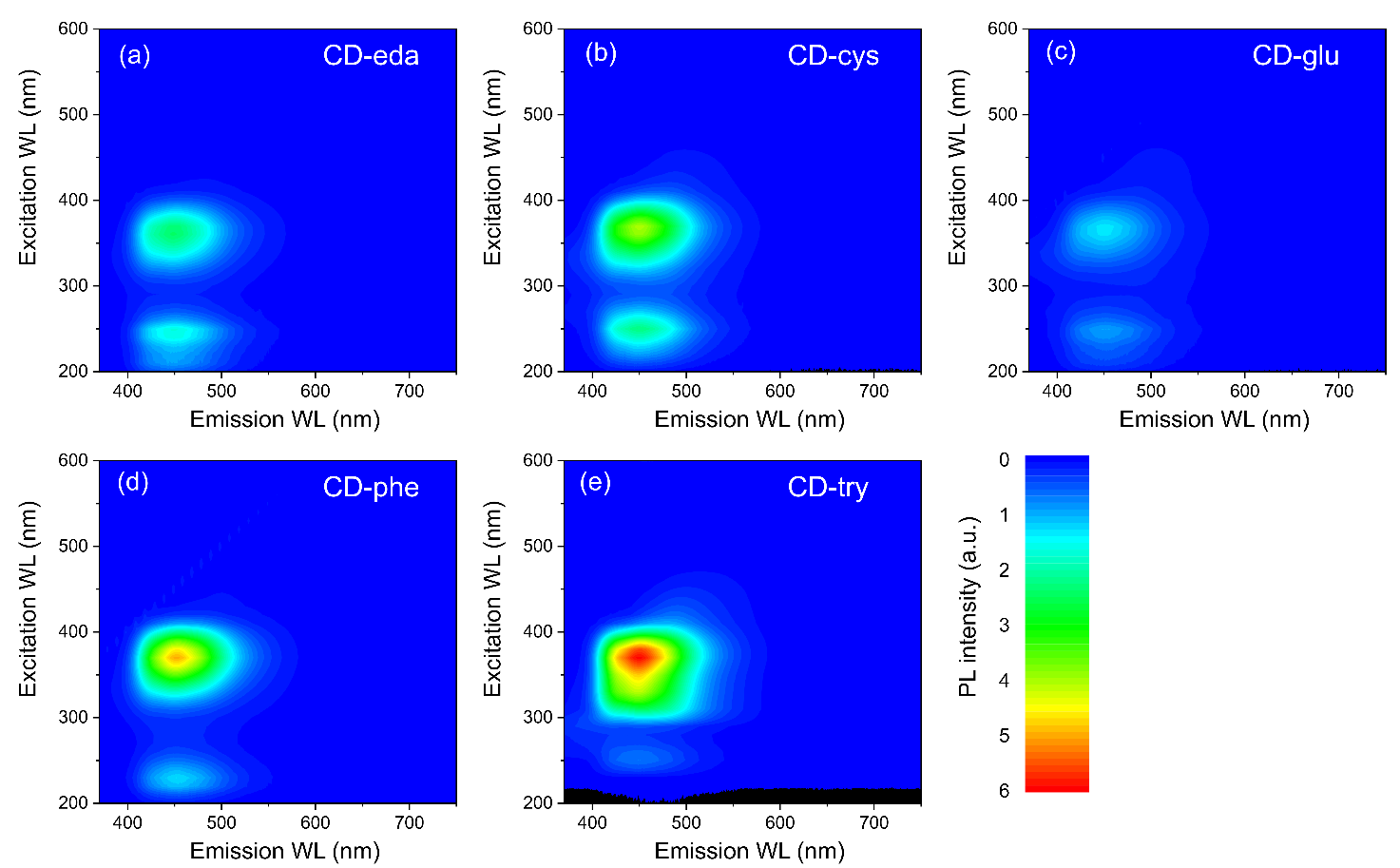


Figure S6. PLE-PL maps of five CD samples: (a) *CD-eda*, (b) *CD-cys*, (c) *CD-glu*_,_ (d) *CD-phe*, and (e) *CD-try*.


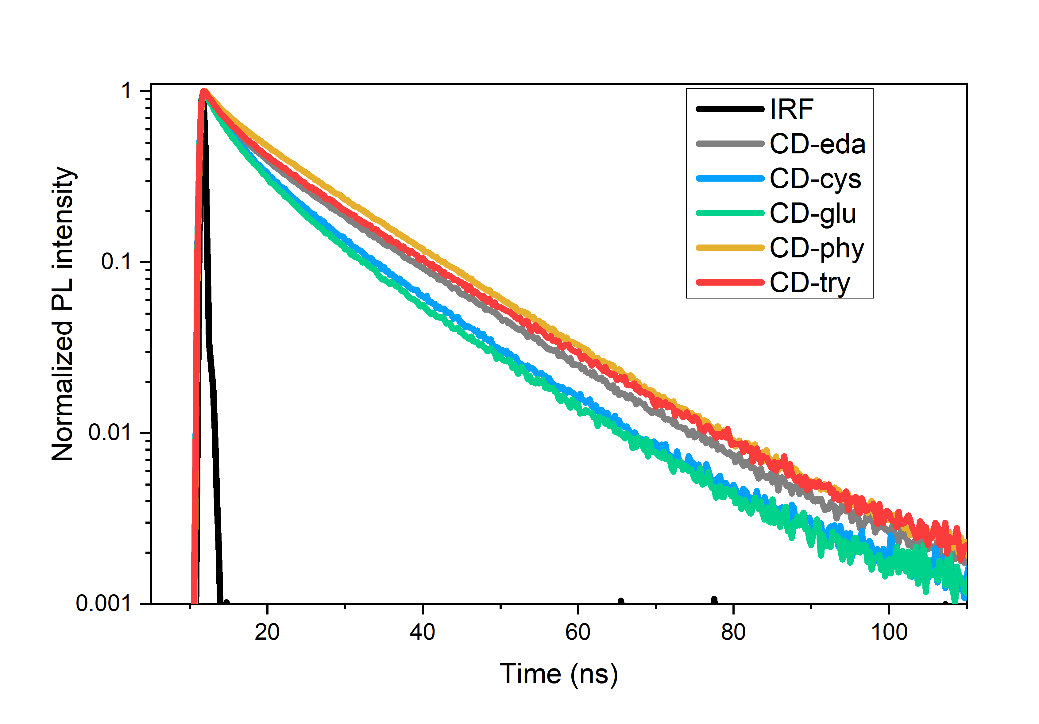


Figure S7. Normalized PL decays of five CD samples: *CD-eda* (grey), *CD-cys* (cyan), *CD-glu* (green), *CD-phe* (orange), *CD-try* (red). Instrument response function (IRF) is shown by a black curve.


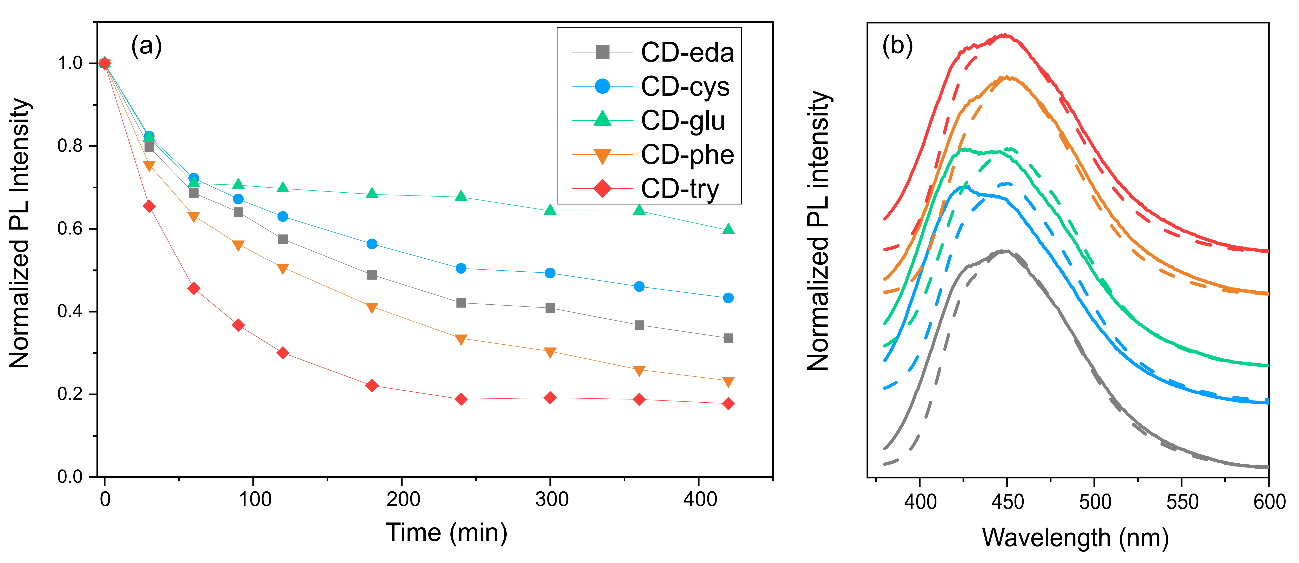


Figure S8. Change of (a) PL intensity and (b) PL profiles of five CD samples before (dashed lines) and after 366 nm UV lamp exposure with a power of 1.4 W·m^-2^ for 420 min (solid lines). The colour coding in (b) is the same as in (a). For chiral *CD-phe*, *CD-try*, and reference sample *CD-eda*, the shorter-wavelength emission was slightly more stable than the longer-wavelength component in their PL spectra, as seen in (b). For *CD-cys* and *CD-glu* the change in the PL band shape was more pronounced, with the longer-wavelength emission quenched more than the shorter-wavelength component in (b).


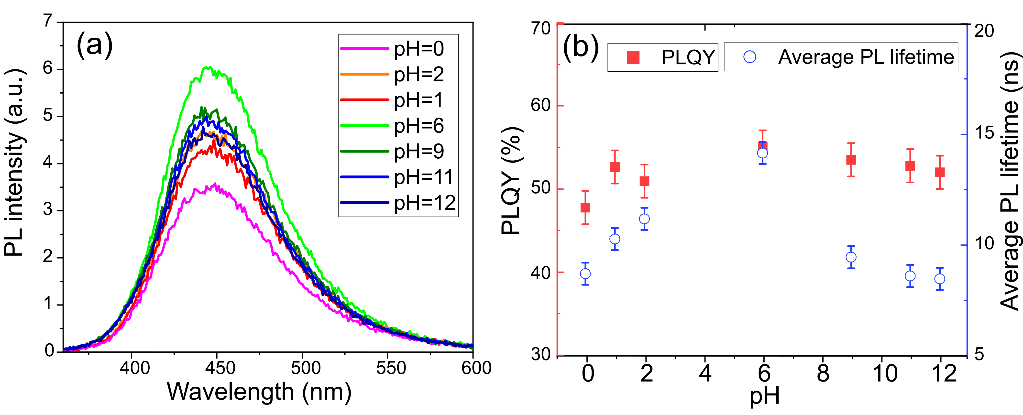


Figure S9. Optical properties of the *CD-phe* as a function of pH. (a) Change of PL spectra (excitation wavelength 350 nm) as a function of pH; (b) PLQY (red squares) and average PL lifetimes (open blue circles) *versus* pH.


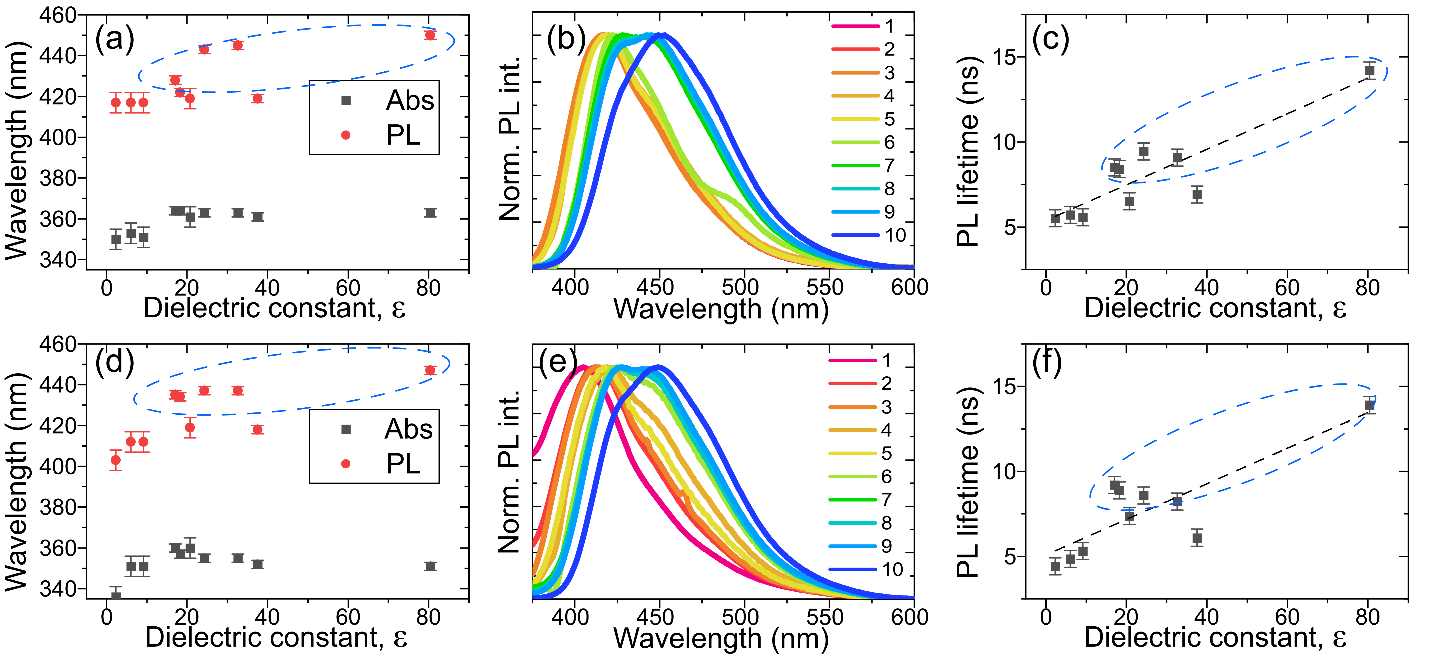


Figure S10. Influence of the solvent polarity effect on optical properties of the *CD-phe* (a-c) and *CD-try* (d-f) dispersed in nonpolar solvents as toluene (1) and chloroform (2), polar aprotic solvents as ethyl acetate (3), acetone (4) and acetonitrile (5), polar protic solvents including alcohols as butanol (6), isopropanol (7), methanol (8) and ethanol (9), and water (10). (a,d) Absorption and PL peak position *versus* solvent dielectric constant ε; (b,e) PL spectra excited at 350 nm; (c,f) average PL lifetime *versus* solvent dielectric constant ε. By dashed blue ellipse the PL peak positions (a,d) and average lifetimes (c,f) for CDs dispersed in polar protic solvents are highlighted. *For CD-phe*, the absorption peak of the band attributed to n-π* transitions was observed at 361-364 nm for polar solvents and it blue-shifted to 350 nm while dispersing CDs in toluene (a). *For CD-try*, similar picture was observed: absorption band attributed to n-π* transitions was centred at 350-360 nm with a blue-shift to 336 nm for CDs dispersed in toluene (d).


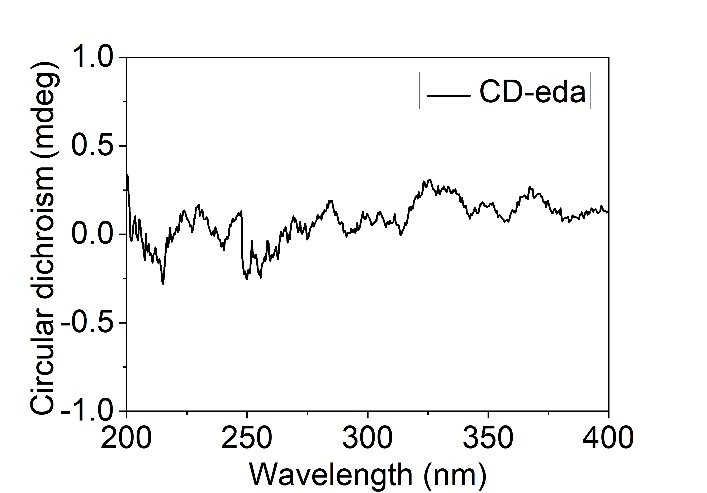


Figure S11. Circular dichroism spectrum of *CD-eda*.


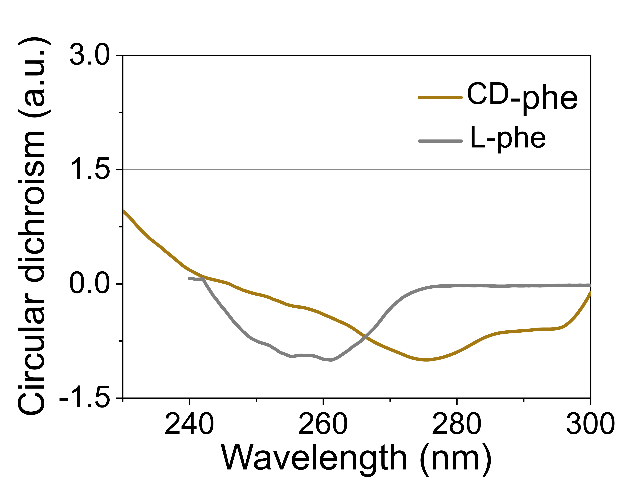


Figure S12. Circular dichroism spectra of *CD-phe* with concentration 2.5 μM and L-phenylglycine (L-phe) with concentration 5 mM.


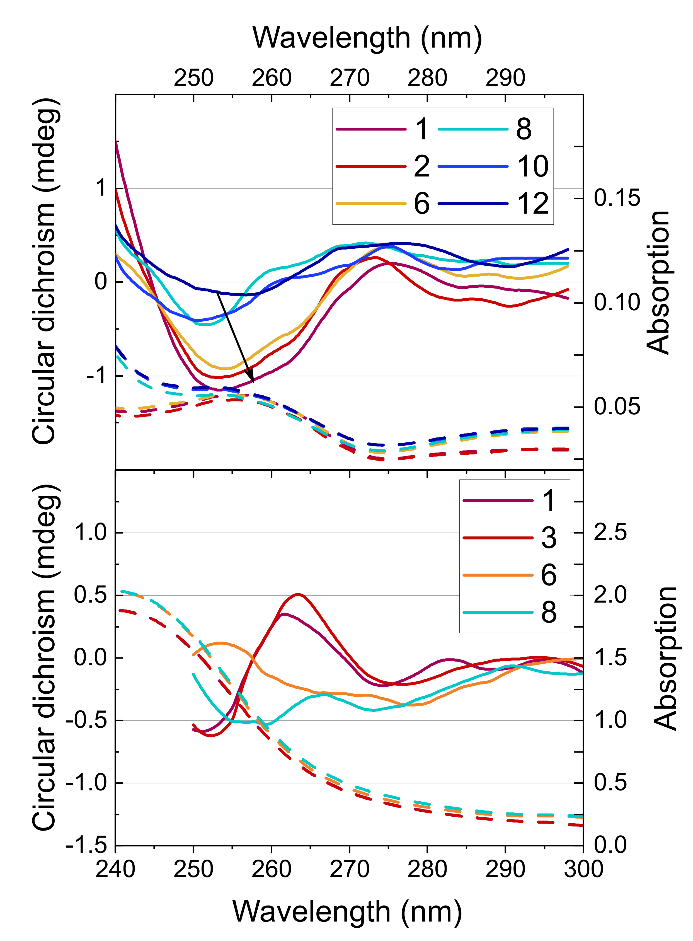


Figure S13. Circular dichroism spectra (solid lines) dependent on pH for L-phenylglycine (L-phe, upper panel) and *CD-phe* (lower panel). The pH values are listed in the legend. Absorption spectra (dashed lines) are shown for comparison. For L-phenylglycine with molar concentration of 2 mM dissolved in water, the amplitude of circular dichroism minimum at 250 nm increases and redshifts to 255 nm with pH decrease (shown by arrow). This indicates that the decrease of pH induces the aggregates formation. For *CD-phe*, the decrease of pH from neutral pH=6 to acid pH=1 results in appearance of additional maximum in circular dichroism spectrum at 265 nm. The increase of pH from pH=6 to pH=8 has almost no impact on circular dichroism signal. Considering that under acid conditions the L-phenylglycine molecules tend to form aggregates, the maximum at 265 nm in *CD-phe* spectrum can be attributed to increase of the aggregative state of L-phenylglycine at the CD’s surface.


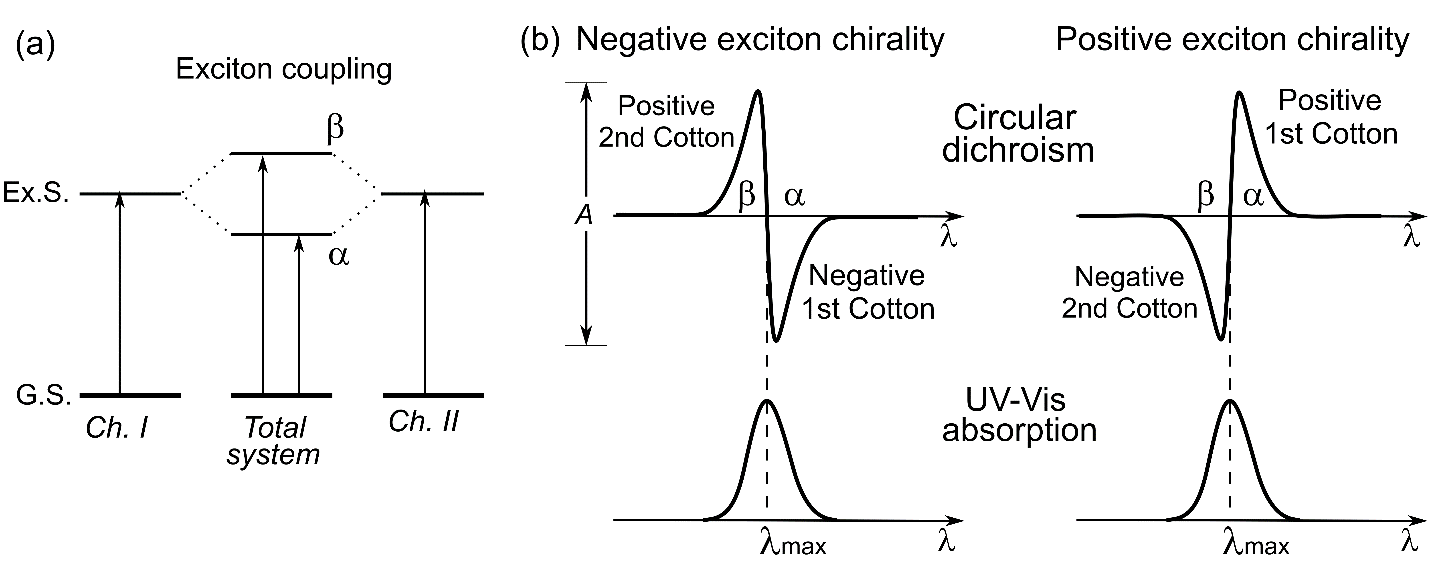


Figure S14. (a) Scheme of exciton coupling of two identical chromophores (*Ch. I* and *Ch. II*) (b) Cotton effect related to absorption band. If the long axes of two interacting chromophores constitute a clockwise screw sense, the circular dichroism spectrum shows a positive first Cotton effect at a longer wavelength and a negative second Cotton effect at a shorter wavelength and is called positive exciton chirality [N. Harada, N. Berova, *Spectroscopic Analysis: Exciton Circular Dichroism for Chiral Analysis*, Elsevier Ltd., 2012].


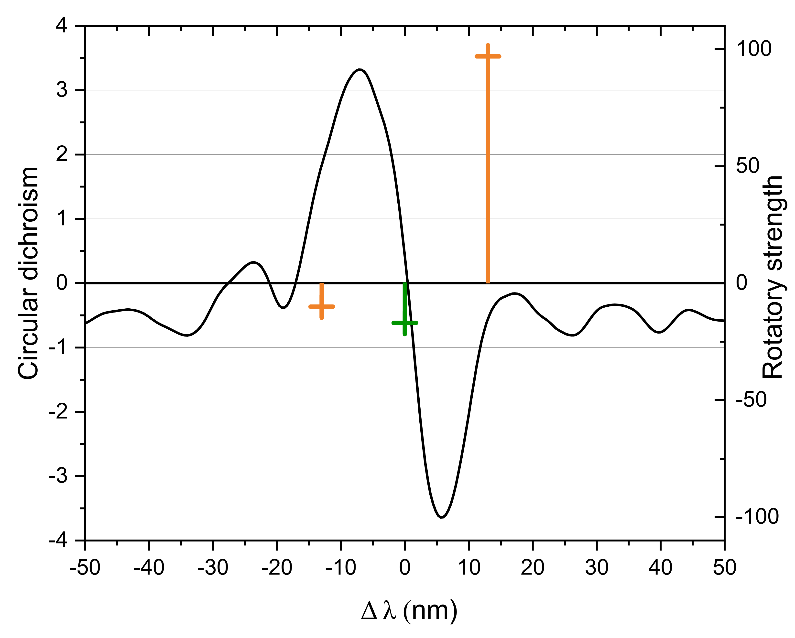


Figure S15. Comparison of calculated rotary strength for n1 (green cross) and n2 (orange crosses), and measured circular dichroism spectrum for *CD-cys* at wavelength scale relative to the maximum of absorption band at 313, 331, and 340 nm for (n1), (n2), and *CD-cys*.


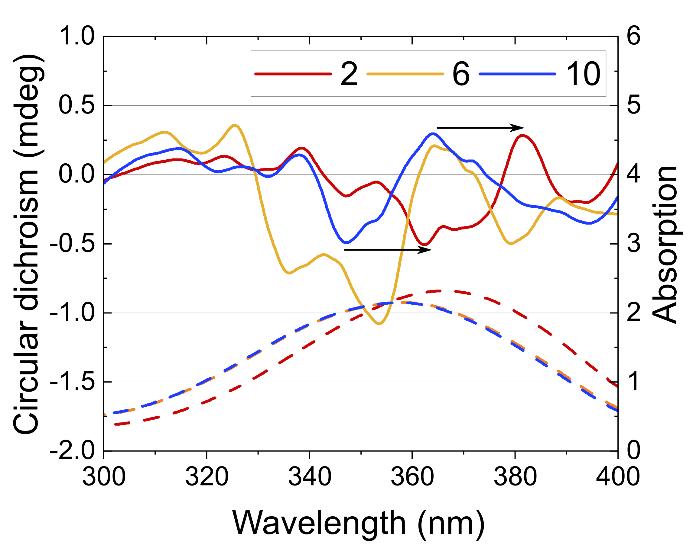


Figure S16. Circular dichroism spectra (solid lines) and absorption spectra (dashed lines) of *CD-phe* dependent on pH. The pH values are listed in the legend. The increase of pH up to pH=10 results in slight decrease in circular dichroism signal (blue line) compared to initial *CD-phe* (yellow line) with no changes in absorption spectrum. The decrease of pH down to pH=2 results in the redshift of bisignate Cotton effects from 352 / 364 nm to 370 / 381 nm for minimum / maximum which agrees well with the redshift of the absorption band from 355 to 367 nm.

Table S1. Chemical composition of five CD samples determined from XPS data

| Sample | C, % | O, % | N, % | S,% |
| --- | --- | --- | --- | --- |
| CD-eda | 55.0 | 28.7 | 16.1 | - |
| CD-cys | 56.6 | 27.1 | 15.8 | 0.5 |
| CD-glu | 56.5 | 27.3 | 15.8 | 0.4 |
| CD-phe | 55.1 | 30.8 | 13.9 | - |
| CD-try | 57.8 | 26.7 | 15.4 | - |

Table S2. Optical characteristics of five CD samples and selected organic dyes: citrazinic acid (*CzA*) and 1, 2, 3, 5-tetrahydro-5-oxo-imidazo [1, 2-a] pyridine-7-carboxylic acid (*IPCA*)

| Sample | n-π*  absorption peak, nm | PL peak, nm (ex@350nm) | PL peak, nm (ex@405nm) | PLQY, % (ex@350nm) | PL lifetime, ns (ex@405nm) | Intrinsic PL lifetime, ns | k_r_ 10^7^, s^-1^ | k_nr_ 10^7^, s^-1^ |
| --- | --- | --- | --- | --- | --- | --- | --- | --- |
| CD-eda | 345 | 450 | 455 | 51 | 13.3 | 26.1 | 3.8 | 3.7 |
| CD-cys | 340 | 450 | 455 | 41 | 11.5 | 28.0 | 3.6 | 5.1 |
| CD-glu | 340 | 450 | 460 | 30 | 10.9 | 36.3 | 2.8 | 6.4 |
| CD-phe | 363 | 450 | 455 | 55 | 14.2 | 25.8 | 3.8 | 3.2 |
| CD-try | 352 | 450 | 455 | 57 | 13.9 | 24.4 | 4.1 | 3.1 |
| Organic dyes | | | | | | | | |
| CzA | 350 | 430* | 470** | 32 | 6.0 | 20.0 | 5.0 | 10.6 |
| IPCA | 350 | 442*** |  | 85.8 | 14.06 | 16.4 | 6.1 | 1.0 |

** excited at 340 nm, ** excited at 400 nm, *** PL peak position is excitation-independent.*

Table S3. Influence of chemical environment on optical characteristics of *CD-phe* in aqueous solution

| pH value | PLQY, % (ex@350nm) | PL lifetime, ns (ex@405nm) | Intrinsic PL lifetime, ns | k_r_ 10^7^, s^-1^ | k_nr_ 10^7^, s^-1^ | k_r_/k_nr_ |
| --- | --- | --- | --- | --- | --- | --- |
| 0 | 48 | 8.8 | 18.3 | 5.5 | 5.9 | 1.1 |
| 1 | 53 | 10.3 | 19.4 | 5.1 | 4.6 | 0.9 |
| 2 | 51 | 11.2 | 21.9 | 4.6 | 4.4 | 1.0 |
| 6 | 55 | 14.2 | 25.8 | 3.9 | 3.2 | 0.8 |
| 9 | 54 | 9.5 | 17.6 | 5.7 | 4.8 | 0.9 |
| 11 | 53 | 8.6 | 16.2 | 6.2 | 5.5 | 0.9 |
| 12 | 52 | 8.5 | 16.3 | 6.1 | 5.6 | 0.9 |

Table S4. Wavelengths ($\lambda$), oscillator strengths ($f$), and rotatory strength ($R$) of the absorption transitions in the longer-wavelength region (300-400 nm) of the naphthol-based surface optical centers of CDs illustrated in Figure 6a.

| Subunit | $\lambda, nm$ | $f$, a.u. | $R$ |
| --- | --- | --- | --- |
| **n1** | 313 | 0.500×10^-1^ | -16.984 |
| **n2** | 318 | 0.347×10^-1^ | -10.024 |
|  | 344 | 0.663×10^-1^ | 96.927 |
| **n3** | 303 | 0.153×10^-1^ | 26.819 |
|  | 309 | 0.357×10^-1^ | 9.6971 |
|  | 319 | 0.115×10^-1^ | -45.496 |
| **n4** | 300 | 0.840×10^-2^ | 14.924 |
|  | 317 | 0.368×10^-1^ | 10.672 |
|  | 321 | 0.311×10^-1^ | -22.369 |
|  | 364 | 0.473×10^-2^ | -10.255 |

Table S5. Calculation of dissymmetry factor (g-factor) for four chiral CD samples

| Sample | Wavelength (nm) | Circular dichroism (Circ.D), mdeg | Optical density for 1 cm cuvette (A) | | g-factor* |
| --- | --- | --- | --- | --- | --- |
| CD-cys | 210 | 2.5 | 1 | 7.6·10^-5^ | |
|  | 240 | -2.4 | 2 | -3.6·10^-5^ | |
|  | 332 | 3.32 | 3.4 | 2.9·10^-5^ | |
|  | 345 | -3.6 | 3.5 | -3.12·10^-5^ | |
| CD-glu | 224 | -1.12 | 0.28 | -1.21·10^-4^ | |
|  | 205 | -3.8 | 0.83 | -1.38·10^-4^ | |
|  | 362 | 3 | 3.5 | 2.6·10^-5^ | |
|  | 383 | -1.46 | 2.6 | -1.7·10^-5^ | |
| CD-phe | 203 | 6.2 | 0.35 | 5.4·10^-4^ | |
|  | 215 | 5.3 | 0.10 | 1.6·10^-3^ | |
|  | 275 | -1.5 | 0.14 | -3.2·10^-4^ | |
|  | 362 | 3.8 | 3.5 | 3.3·10^-5^ | |
|  | 352 | -4.6 | 3.4 | -4.1·10^-5^ | |
| CD-try | 209 | -1.14 | 1.04 | -3.32·10^-5^ | |
|  | 218 | 1.15 | 1.05 | 3.32·10^-5^ | |
|  | 345 | 3.32 | 3.46 | 2.9·10^-5^ | |
|  | 356 | -2.03 | 3.43 | 1.8·10^-5^ | |

**g-factor has been calculated using the following equation: G = Circ.D (in mdeg) / (A·32980), where A is the optical density for 1 cm cuvette.*

Table S6. Estimation of two-photon absorption cross-sections for five CD samples, compared with Rhodamin 6G dye.

|  | Concentration, µM | Optical density @400nm | ε @400nm ×10^5^ | PLQY, % | Int TPL, a.u. | σ, GM |
| --- | --- | --- | --- | --- | --- | --- |
| CD-eda | 2.48 | 0.169 | 1.36 | 56 | 9411 | 57 |
| CD-cys | 4.56 | 0.156 | 0.68 | 47 | 9318 | 44 |
| CD-glu | 2.48 | 0.153 | 1.23 | 28 | 5834 | 141 |
| CD-phe | 4.97 | 0.164 | 0.66 | 75 | 17214 | 29 |
| CD-try | 2.9 | 0.142 | 0.98 | 59 | 8267 | 39 |
| Rhodamin 6G | 15.8 |  |  | 90 | 338664 | 33 |
